# Marine Cyanobacteria: A Rich Source of Structurally Unique Anti-Infectives for Drug Development

**DOI:** 10.3390/molecules29225307

**Published:** 2024-11-10

**Authors:** Lik Tong Tan, Nurul Farhana Salleh

**Affiliations:** Natural Sciences and Science Education, National Institute of Education, Nanyang Technological University, 1 Nanyang Walk, Singapore 637616, Singapore; farnaaa93@gmail.com

**Keywords:** marine cyanobacteria, anti-infectives, antibacterial, quorum sensing modulators, antifungal, antiviral, antiparasitic

## Abstract

Marine cyanobacteria represent a promising yet underexplored source of novel natural products with potent biological activities. Historically, the focus has been on isolating cytotoxic compounds from marine cyanobacteria, but a substantial number of these photosynthetic microorganisms also produce diverse specialized molecules with significant anti-infective properties. Given the global pressing need for new anti-infective lead compounds, this review provides a concise yet comprehensive overview of the current knowledge on anti-infective secondary metabolites derived from marine cyanobacteria. A majority of these molecules were isolated from free-living filamentous cyanobacteria, while several examples were derived from marine cyanobacterial symbionts. In addition, SAR studies and potent synthetic analogs based on selected molecules will be featured. With more than 200 molecules, this review presents their antibacterial, antifungal, antiviral, antiprotozoal, and molluscicidal activities, with the chemical and biological information covered in the literature up to September 2024.

## 1. Introduction

The global burden of infectious diseases remains one of the most pressing challenges in public health, driving an urgent need for the development of effective anti-infective agents [1]. These agents, including antivirals, antibacterials, and antiparasitics, play a critical role in combating the wide array of pathogens responsible for infections in humans. For instance, the rise of antimicrobial resistance (AMR) poses a significant threat to the efficacy of existing treatments, making the search for novel therapeutics more crucial than ever [2]. In addition to the traditional bacterial, parasitic, and viral pathogens, emerging and re-emerging infectious diseases further underscore the necessity for a robust arsenal of anti-infective drugs.

Antimicrobials have revolutionized medicine since their discovery, substantially reducing the morbidity and mortality associated with bacterial infections [3]. However, the widespread and often inappropriate use of these drugs has led to the evolution of resistant strains, diminishing their effectiveness. Similarly, antiparasitic agents have been vital in controlling diseases such as malaria, yet resistance to these medications is also on the rise, threatening global health efforts. Viral infections, too, present a significant challenge, with diseases such as HIV, influenza, and, more recently, COVID-19 highlighting the critical need for effective antiviral therapies [4].

Marine cyanobacteria, also known as blue-green algae, are ancient microorganisms that have adapted to thrive in diverse marine environments, including coral reefs and the open ocean. This adaptability has driven the evolution of unique metabolic pathways, resulting in the production of a wide array of secondary metabolites [5]. These metabolites, while not essential for the basic metabolic processes of cyanobacteria, play crucial roles in their survival and interaction with the environment, including defense mechanisms against predators and competitors [6]. The unique chemical diversity and biological activities of marine cyanobacterial secondary metabolites have garnered significant attention due to the pioneering work by Richard E. Moore on bioactives from marine cyanobacteria. These compounds have shown promise in a variety of therapeutic areas, including antimicrobial, antiviral, anticancer, and anti-inflammatory applications [7]. Research has demonstrated that cyanobacterial secondary metabolites often possess novel structures and mechanisms of action that are distinct from those found in terrestrial organisms [7]. This structural novelty is particularly valuable in the context of drug discovery, as it provides new templates for the development of therapeutics with potentially improved efficacy and reduced resistance. For instance, marine cyanobacteria have been found to produce compounds with potent activity against multidrug-resistant bacteria, as well as novel anticancer agents that target specific cellular pathways. The increasing prevalence of drug-resistant pathogens, coupled with the ongoing search for novel and effective treatments for cancer and other diseases, underscores the importance of exploring these marine natural products as potential drug agents.

This comprehensive review article aims to highlight the importance of marine cyanobacterial secondary metabolites as sources of potential anti-infective agents, including antiviral, antibacterial, and antiparasitic molecules. As long as there are reports of antimicrobial and antiparasitic properties associated with marine cyanobacterial compounds, they will be included in our discussion. This is to provide information on the range of structurally diverse compounds having anti-infective activities that could spur their further research. As a result, more than 200 molecules are included in this review, and they are presented based on their biological activities (Table 1). This review will explore the current state of research in the field, including molecular targets of promising anti-infective metabolites and their identified synthetic analogs. Selected molecules having different anti-infective activities with high potency will be discussed in their respective sections. Examples of such molecules include dolastatin 10 (antifungal and antimalarial activities), gallinamide A (antiviral and antiparasitic activities), and anaephenes (antibacterial and antiparasitic activities). In some cases, it is the specific mode of action of compounds, such as the potent human cathepsin L-inhibitory activity of gallinamide A, that facilitates their further exploration in other anti-infective area. By shedding light on the untapped potential of marine filamentous cyanobacteria, this article seeks to emphasize the need for continued exploration and investment (e.g., their synthesis and further biological evaluation) in this area, which holds the promise of discovering new, life-saving drugs for a variety of infectious diseases.

## 2. Antivirals

Viruses exhibit a wide range of life cycles, intricately intertwined with those of their hosts, making the development of effective antiviral treatments a highly challenging endeavor. For instance, viruses like the human immunodeficiency virus (HIV), hepatitis C, dengue, herpesviruses, Ebola, and the more recent coronaviruses can impact a significant portion of the global population. While immunotherapeutics can be highly effective, their development is often complex and expensive, which has spurred interest in non-immunogenic alternatives. A wide range of antiviral cyanobacterial compounds, including peptides, cyanobacterial lectins, polysaccharides (e.g., sulfated polysaccharides), sulfoglycolipids, polyketides, and alkaloids, have been reported [113]. This section provides a selection of low-molecular-weight antiviral molecules isolated from marine cyanobacteria.

### 2.1. Aplysiatoxins

**Figure 1:**
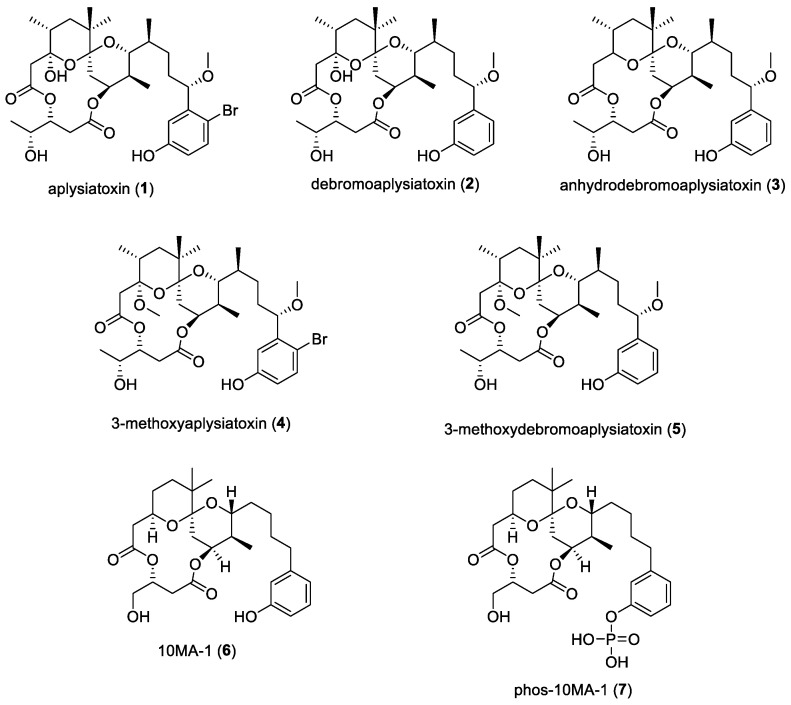
Structures of natural and synthetic aplysiatoxin-related molecules.

Polyketide-derived aplysiatoxins are a group of marine toxins that have been investigated for their antiviral properties and have been isolated from several filamentous marine cyanobacterial species, including *Lyngbya majuscula*, *Schizothrix calcicole,* and *Oscillatoria nigro-virdis* [114]. Aplysiatoxin (**1**) and debromoaplysiatoxin (**2**) (Figure 1) were originally discovered from a marine gastropod mollusk in 1975 [115]. In 2014, they were isolated from the marine cyanobacterium *Trichodesmium erythraeum*, along with anhydrodebromoaplysiatoxin (**3**), 3-methoxyaplysiatoxin (**4**), and 3-methoxydebromoaplysiatoxin (**5**), collected on Seringat Island, Singapore (Figure 1) [8]. These compounds were tested against the Chikungunya virus (CHIKV), which is a mosquito-transmitted virus, in the same study. It was found that the debrominated compounds exhibited a dose-dependent inhibitory activity against CHIKV-infected cells. In particular, debromoaplysiatoxin (**2**) and 3-methoxydebromoaplysiatoxin (**5**) were potent against CHIKV with EC_50_ values of 1.3 μM and 2.7 μM and selectivity indices of 10.9 and 9.2, respectively. It was also hypothesized that these compounds possibly targeted steps in the replication cycle of the virus that occur after viral entry, making them a novel class of antiviral agents.

Aplysiatoxins were also studied for activity against HIV-1. Novel therapeutics were required to fight against HIV-1 as there were latent HIV-1-infected cells on which traditional strategies were found to be ineffective. One such strategy, called “shock-and-kill”, utilizes latent-reversing agents (LRAs) to induce proviral expression (“shock”), followed by the termination of these cells by viral cytopathic effects or the host immune response [116]. These LRAs were found to either be histone deacetylase inhibitors (HDACis) or protein kinase C (PKC) activators [117]. Aplysiatoxin (**1**), a known PKC activator, was found to have the ability to induce proviral expression up to 900-fold-lower concentrations compared to prostatin and without substantial effects on cell viability [9].

A synthetic analogue of aplysiatoxin, 10-methyl-aplog-1 (10MA-1) (**6**) (Figure 1), combined with a bromodomain and extra-terminal motif inhibitor, JQ1, was found to efficiently and synergistically reactivate proviral expression [10]. Using 10MA-1 is more attractive than prostatin as its chemical synthesis is simpler, making 10MA-1 advantageous. However, water solubility issues were found for 10MA-1, which led to dose limitations in vivo. Another study synthesized a phosphate ester of 10MA-1, 18-O-phospho-10-methyl-aplog-1 (phos-10MA-1) (**7**) (Figure 1), to overcome this issue. Although its activities were found to be similar to 10MA-1 in vitro, further investigation was needed in the form of in vivo experiments to optimize its metabolic stability [11].

### 2.2. Serinol-Derived Malyngamides

**Figure 2:**
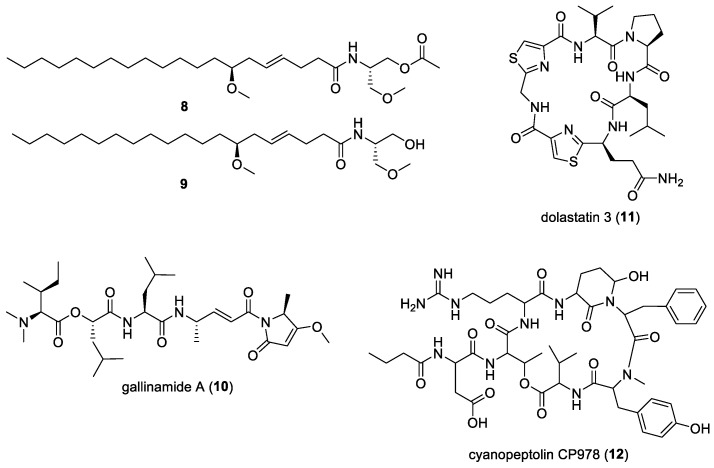
Structures of antiviral marine cyanobacterial molecules.

Two malyngamide analogs, (**8**) and (**9**) (Figure 2), which are serinol-derived malyngamides, were isolated from an Australian cyanobacterium. Unfortunately, attempts to identify this microalgal specimen have not been successful. These compounds were classified as malyngamides due to their fatty acid residues, with differences in their amino acid residues, and they were simpler than previously discovered malyngamides. When evaluated at NCI, the two compounds were found to exhibit weak anti-HIV activity [12].

### 2.3. Gallinamide (= Symplostatin 4)

Gallinamide A (**10**) (Figure 2) was first isolated from the marine cyanobacterium *Schizothrix* sp. in 2009 and exhibited potent antimalarial activities [13]. The subsequent total synthesis of gallinamide A confirmed that its structure and stereochemistry are identical to antimalarial symplostatin 4 [118,119]. Further investigations proved that gallinamide A (**10**) was also a potent human cathepsin L inhibitor [14], key to antiviral activities, including the inhibition of SARS-CoV-2 [15]. In recent studies, gallinamide A and its synthetic analogs were found to be effective inhibitors of cathepsin L, with IC_50_ values in the low nanomolar to picomolar range. The mechanism of gallinamide A involved the covalent inhibition of the host cysteine protease cathepsin L, playing an important role in viral entry [16]. Due to its potency, the total synthesis of gallinamide A was carried out for pre-clinical studies against COVID-19. This resulted in the successful second-generation total synthesis of gallinamide A with a yield of 315 mg over 16 steps, which was 32% percentage yield [120].

### 2.4. Dolastatin 3

Dolastatin 3 (**11**) (Figure 2) was first isolated from the sea hare *Dolabella auricularia* [121] and identified as an anticancer compound. In 2000, the same compound was isolated from the filamentous marine cyanobacterium *Lyngbya majuscula*, collected from a lagoon near the Big Goby marine lake, in Palau, and was found to be effective against HIV-1-infected cells based on HIV-1 integrase inhibition assays [18]. Along with dolastatin 3, a closely related compound, homodolastatin 3, was isolated and found to be ineffective against HIV-1. The only difference between the two compounds is the replacement of the valine residue by isoleucine, which shows that the substituent plays an important role in biological activities. However, dolastatin 3 was not further pursued as an integrase inhibitor due to the loss of molecules during transfers in assays. Due to the potency of dolastatin 3, its total synthesis was conducted [122]. The resulting synthetic (-)-dolastatin 3 was identical to the natural product and was realized in 41% overall yield.

### 2.5. Cyanopeptolins

As part of efforts to find therapeutics for SARS-CoV-2, which had many variants with differences in their transmission, severity, and public health impacts, posing a challenge which needed to be tackled worldwide, cyanobacterial compounds were explored [20]. Subsequently, fifteen cyanopeptolins were isolated from cultures of the Baltic marine cyanobacterium *Nostoc edaphicum* CCNP1411. Among these compounds, the Arg-containing cyanopeptolin CP978 (**12**) (Figure 2) was found to be effective against three SARS-CoV-2 variants—Alpha, Micron, and Delta—with the strongest inhibition of Delta SARS-CoV-2 infection in A549ACE2/TMPRSS2 cells. It was also found that cyanopeptolin CP978 exhibited direct interaction with virions and caused a significant decline in viral replication in primary human airway epithelial cells. These results display the capability of cyanopeptolin CP978, making it an interesting candidate for antiviral therapeutics which should be further explored.

### 2.6. Divamides

Symbiotic relationships between bacteria and their hosts could pose a challenge that many researchers face when characterizing the discovery of natural products, despite the potential that these small molecules possess. One example of this is the studied symbiotic relationship of tunicates and cyanobacteria of the genus *Prochloron*, and, even after more than 40 years of study, it remains uncultivated [123]. Investigating these symbionts is interesting as they are known to be producers of active secondary metabolites and toxins and able to function as chemical defenses to protect themselves and the host from predation.

**Figure 3:**
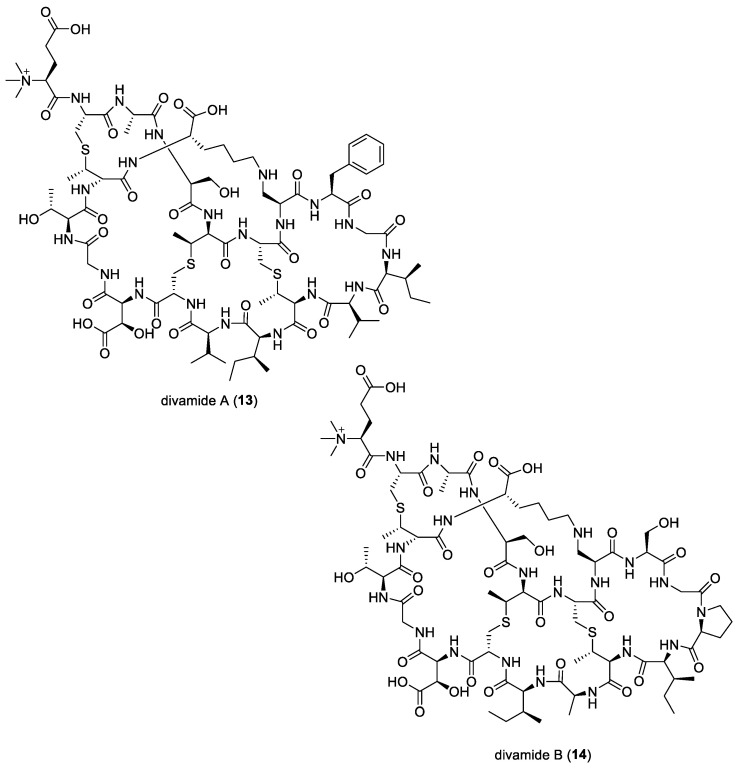
Structures of divamides A and B.

In a study by Smith and co-workers, additional RiPP molecules have been discovered within *Prochloron didemni* cyanobionts found in two neighboring *Didemnum molle* tunicates, collected in Eastern Papua New Guinea. Two anti-HIV compounds from novel family of lanthipeptides, named divamides A (**13**) and B (**14**) (Figure 3), were isolated and investigated with the help of metagenomics, chemistry, and synthetic biology [21]. Divamide A (**13**) was found to contain three methyllanthionines, one lysinoalanine, β-hydroxy aspartic acid, and *N*-terminal trimethylation, which is a naturally rare post-translational modification. It was found, from biosynthetic clusters, that these compounds were synthesized from the symbiont cyanobacterium *Prochloron didemni*. The strategy implemented in the above study was the first to overcome the limitations of supply while studying the secondary metabolites produced by symbiont marine cyanobacteria. It proved to be an efficient strategy when working with a limited resource, as it was not only able to analyze the structure of the compound, but it also allowed for structure–activity relationship investigations with biological testing.

## 3. Antibacterials and Modulators of the Bacterial Quorum Sensing System

The need for new antibacterial drugs is critical due to the rapid emergence of antibiotic-resistant bacteria [124]. The overuse and misuse of existing antibiotics have accelerated the development of resistant strains, rendering many current treatments ineffective. This poses a significant threat to global public health, as infections that were once easily treatable are becoming harder to manage. Additionally, the slow pace of novel antibiotic development further exacerbates the problem, highlighting the urgent need for new drugs to combat resistant pathogens and prevent a future where common infections could once again become deadly. This section highlights various antibacterial molecules, from marine cyanobacteria, including molecules which interfere with the bacterial quorum sensing system.

### 3.1. Antibacterials

#### 3.1.1. 2-Hydroxyethyl-11-hydroxyhexadec-9-enoate

**Figure 4:**
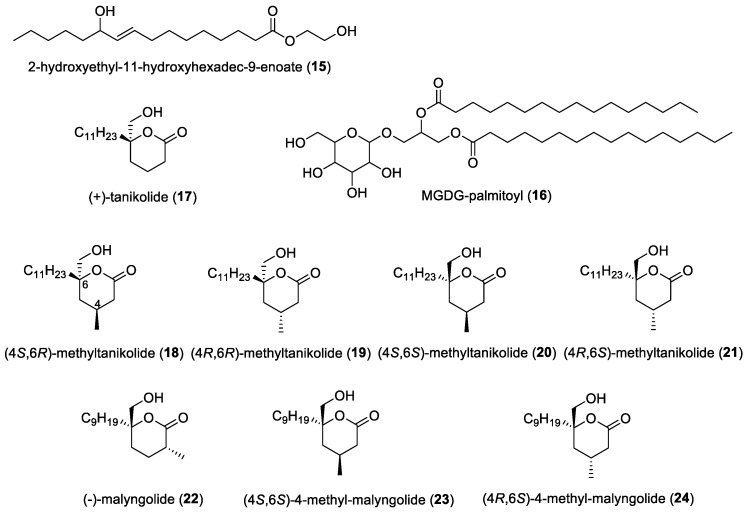
Antimicrobial marine cyanobacterial compounds and synthetic analogs.

Isolated from *Leptolyngbya* sp. LT19, the novel antibacterial compound 2-hydroxyethyl-11-hydroxyhexadec-9-enoate (**15**) (Figure 4) was discovered by Maneechote et al. and found to exhibit activities against the Gram-negative shrimp pathogens *Vibrio harveyi* and *V. parahaemolyticus* [22]. Biological assays showed that it exhibited minimal inhibitory concentrations of 250–1000 and 350–1000 µg/mL, respectively. These values indicated that the compound was more active than ampicillin and penicillin, which showed minimum inhibitory concentrations of 500 and 800 µg/mL, respectively. This activity was found to be similar to the activity of long-chain fatty acids, such as oleic acid [125]. This secondary metabolite could prove its significance in the shrimp industry due to its high activity against *Vibrio* sp. It is also possible for this novel compound to be used as an alternative to control vibriosis, a common disease which poses a threat to worldwide shrimp production.

#### 3.1.2. Monogalactosyldiacylglycerol (MGDG) Containing a Palmitoyl

Extended-spectrum β-lactamase (ESBL)-producing bacteria pose a serious threat in clinical settings and are linked to high rates of morbidity and mortality [126]. This makes it important for researchers to discover and develop new antibacterial compounds to overcome bacterial resistance. In a study by Ahamed et al., monogalactosyldiacylglycerol containing a palmitoyl (MGDG-palmitoyl) (**16**) (Figure 4) was discovered from the marine cyanobacterium *Oscillatoria acuminata* NTAPC05 through a bioassay-guided fractionation of its methanol extract [23]. The cyanobacterial samples were collected from Mandapam, Ramanathapuram District, Tamil Nadu, India. The fractions were tested against three ESBL-producing bacteria stains, including *Escherichia coli* U655, *Enterobacter asburiae* B938, and *Stenotrophomonas maltophilia* B929, which were extensively characterized for the study in question. MGDG-palmitoyl (**16**)’s composition was elucidated through extensive spectral analyses and was found to be the first MGDG compound to contain a palmitoyl that exhibited activity against ESBL producers. Based on confocal laser scanning microscopy analyses, this active molecule appears to damage the bacterial membrane, leading to the lysis of bacterial cells.

#### 3.1.3. Tanikolide, Malyngolide, and Related Analogs

*Staphylococcus aureus* is a bacterium that was reported to be among the most aggressive human pathogenic agents, posing a major challenge to clinical practice [127]. It is therefore important for scientists to investigate potential candidates of chemical compounds to overcome this challenge. Biological activities of (+)-tanikolide (**17**) and its analogs, including **18**–**21** (Figure 4), were investigated by Breheny et al. against methicillin-resistant *Staphylococcus aureus* [24]. The natural tanikolide was originally reported by Gerwick and co-workers as a brine shrimp toxic and antifungal molecule from the marine cyanobacterium *Lyngbya majuscula*, obtained from Tanikeli Island, Madagascar [25]. Tanikolide analogs **18**–**21** were tested against *Escherichia coli* and methicillin-resistant *Staphylococcus aureus* (MRSA), and it was revealed that (4*S*,6*S*)-4-methyltanikolide (**20**) showed potential against MRSA with a minimum inhibitory concentration of 12.5 µg/mL [24], comparable to that of vancomycin and linezolid. It was found that the configuration of the methyl group to the carbonyl resulted in this activity, compared to (4*R*,6*S*)-4-methyltanikolide (**21**), which did not show any activity. No antibacterial activity against *Escherichia coli* was found among these four tanikolide analog compounds.

Malyngolide (**22**) (Figure 4), which was first isolated by Cardellina et al. [26], originated from the lipid extract of the filamentous marine cyanobacterium *Lyngbya majuscula*, from Kahala Beach, Oahu. This compound was found to be active against *Mycobacterium smegmatis* and *Streptococcus pyogenes* and less active against *Staphylococcus aureus* and *Bacillus subtilis*. It was reported to be inactive against several other bacterial strains, including *Enterobacter aerogenes*, *E. coli*, *Pseudomonas aeruginosa*, *Salmonella enteritidis,* and *Staphylococcus marcescens*. In a study by Breheny et al., two analogs of malyngolide (**23** and **24**) (Figure 4) were synthesized and investigated, and it was found that the adjustment of the n-nonyl alkyl side chain showed an increase in minimum inhibitory concentrations when tested against MRSA to 50 µg/mL compared to (4*S*,6*S*)-4-methyltanikolide (**20**) [24].

#### 3.1.4. Anaephenes

**Figure 5:**
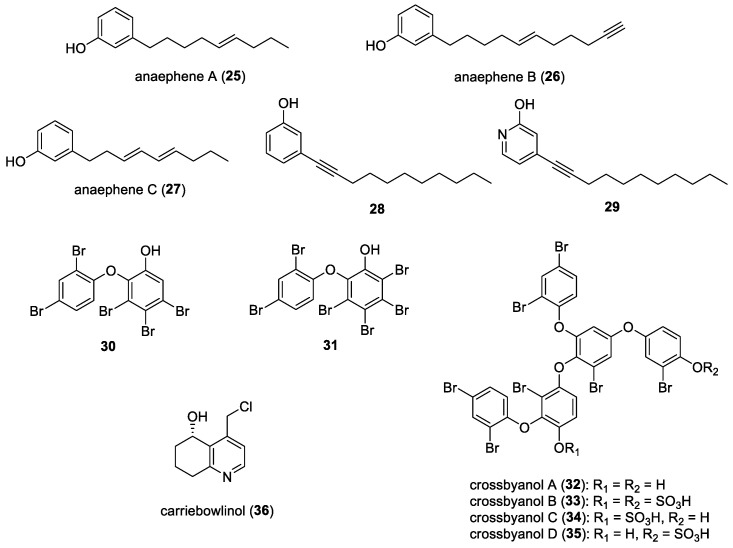
Antimicrobial marine cyanobacterial compounds and synthetic analogs.

Anaephenes A (**25**)–C (**27**) (Figure 5) are alkylphenols which were first isolated from the filamentous marine cyanobacterium *Hormoscilla* (Oscillatoriales) from Anae Island, Guam [30]. Anaephene B, which contains an alkyne chain, was found to have moderate activity against the bacterial strains *Bacillus cereus* and *Staphylococcus aureus,* with a minimum inhibition concentration of 6.1 µg/mL. There are reports on the total synthesis of anaephenes and analogs to investigate the structure–activity relationship of their antibacterial activities. Kukla et al. synthesized anaephenes A (**25**) and B (**26**), and further testing found that each of these compounds exhibited antibacterial activities against MRSA with minimum inhibitory concentrations of 16 µg/mL and 8 µg/mL, respectively [31]. In another study by Kukla et al. [32], the structures of these two alkyphenols were further adjusted at the phenol moiety and the alkyl chains, producing 18 analogs, to investigate the effect on MRSA. It was found that, with an internal alkyne instead of a terminal alkyne, such as compound **28** (Figure 5), the minimum inhibitory concentration against MRSA improved to 2 µg/mL, which was 4-fold more potent than anaephene B, and 2-fold more potent than the FDA-approved antibiotic linezolid which was used as a positive control in the experiments. Another analogue, containing a 2-hydroxypyridine moiety, compound **29** (Figure 5), exhibited a minimum inhibitory concentration of 8 µg/mL against MRSA, making it as potent as anaephene B.

#### 3.1.5. Polybrominated Diphenyl Ethers

Natural polybrominated diphenyl ethers (PBDEs) are commonly found in marine sponges, especially sponges of the order Dysideidae, but it was revealed that the true source of these compounds lies in their biosynthesis by symbiotic cyanobacteria [128]. Using metagenomic approaches, the biosynthetic gene clusters of PBDEs were found to originate from the cyanobacterial symbiont *Hormoscilla spongeliae* (formerly *Oscillatoria spongeliae*) [34]. PBDEs were first isolated from marine sponges of the *Dysidea* species in 1981, and they possess diverse biological activities, including anticancer, antibacterial, and antifungal properties. For instance, several PBDEs have been recently reported to have antibacterial properties, including the known 3,4,5-tribromo-2-(2′,4′-dibromophenoxy)phenol (**30**) and 3,4,5,6-tetrabromo-2-(2′,4′-dibromophenoxy)phenol (**31**) (Figure 5), isolated from the Indonesian marine sponge *Lamellodysidea herbacea* [35]. Compound **30** showed stronger inhibition against human pathogenic bacteria and fungi, while compound **31** showed more potent inhibition of the Gram-negative bacterium *Rhodotorula glutinis,* with a minimum inhibitory concentration of 2.1 μg/mL.

#### 3.1.6. Crossbyanols

Isolated from the filamentous marine cyanobacterium *Leptolyngbya crossbyana* found on Hawaiian coral reefs, crossbyanols A (**32**)–D (**35**) (Figure 5) were analyzed through spectroscopy-guided fractionation [36]. These compounds are hepta-brominated polyphenolic ethers, and crossbyanol B (**33**) was found to exhibit antibiotic activity against MRSA with a minimum concentration inhibition of 2.0–3.9 µg/mL and a relatively potent brine shrimp toxicity with an IC_50_ value of 2.8 ppm. It was noted that the two sulfate groups in crossbyanol B could potentially be contributing to these biological activities.

#### 3.1.7. Carriebowlinol

**Figure 6:**
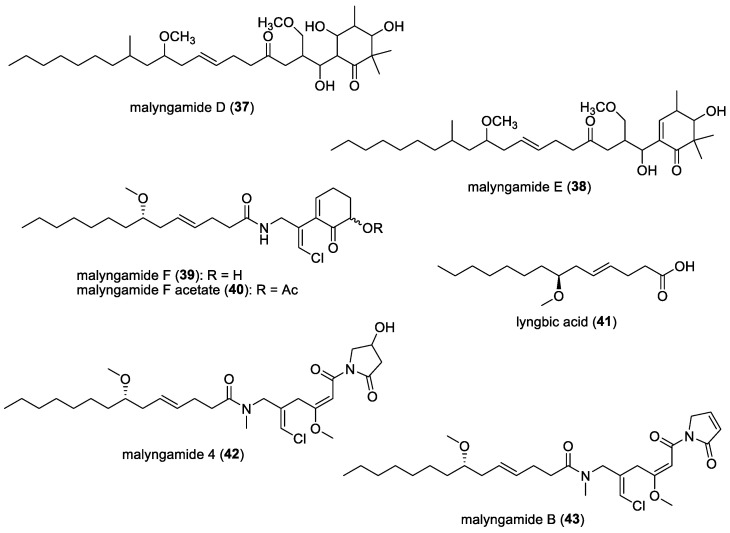
Structures of malyngamide-related compounds and lyngbic acid.

Cyanobacteria having antimicrobial properties are not only crucial for human diseases or infections but also for ecological systems [129]. One such antimicrobial agent is carriebowlinol (**36**) (Figure 5), which was isolated, along with lyngbic acid (**41**) (Figure 6), from a marine filamentous cyanobacterium that was found to be closely related to *Lyngbya majuscula* [37]. These compounds were tested against three species of harmful saprophytic marine fungi, namely *D. salina*, *L. thalassiae,* and *Fusarium* sp. Both compounds, at their isolated concentration, were shown to completely inhibit the growth of all three fungal strains. In addition, carriebowlinol was, on average, 9- to 10-fold more active than lyngbic acid. Furthermore, carriebowlinol exhibited strong antibacterial activity against 11 marine bacterial strains. This indicated potential for carriebowlinol and lyngbic acid as chemical defensive agents in ecological systems, such as their hinderance of microbial biofilm formation.

#### 3.1.8. Malyngamides and Lyngbic Acid

There have been several discoveries of antibacterial compounds belonging to the malyngamide class of molecules, with the first series of malyngamides A-C isolated from *Lyngbya majuscula* by Cardellina and co-workers [130,131] in 1978. For instance, malyngamides D (**37**) and E (**38**) (Figure 6), obtained from deep-water *L. majuscula*, were found to have mild antibiotic activity against *Mycobacterium smegmatis* and *B. suhtilis* [38]. In 1987, two additional malyngamides, malyngamide F (**39**) and malyngamide F acetate (**40**) (Figure 6), were reported from the same species of filamentous marine cyanobacterium, *Lyngbya majuscula* [39]. Malyngamide F was found to have some activity against *Staphylococcus aureus*. In addition, a free methoxylated fatty acid, lyngbic acid (**41**) (Figure 6), isolated together with malyngamides F and F acetate, displayed antimicrobial activity against *Staphylococcus aureus* and *Bacillus subtilus*. Lyngbic acid was also reported to have the highest inhibitory activity against *Mycobacterium tuberculosis* H37Rv at a concentration of 12.5 μg/mL, whereas malyngamide 4 (**42**) (Figure 6), which was discovered by Shaala et al., and the known malyngamide B (**43**) (Figure 6) showed weaker inhibition of mycobacterial growth at the same concentration [40]. Malyngamides are still being discovered; the latest was reported in 2017 by Sueyoshi et al. [132]. Although malyngamides and their related compounds have not been tested extensively for antibacterial activities, they still hold potential in various biological activities, and further biological investigations are warranted.

#### 3.1.9. Pitipeptolides

**Figure 7:**
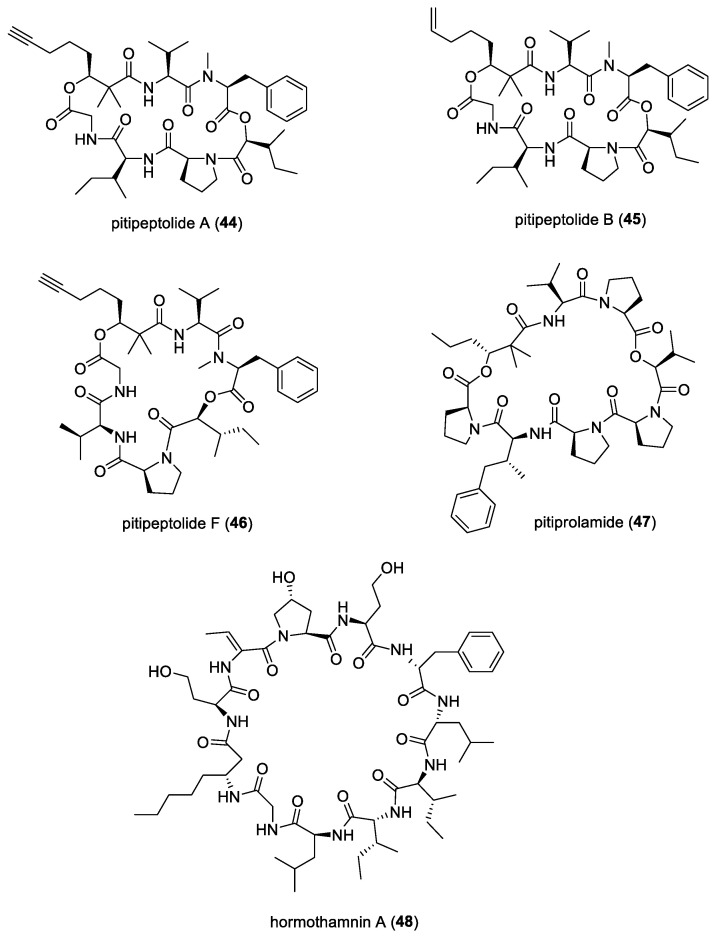
Antibacterial marine cyanobacterial compounds.

Pitipeptolides A (**44**) and B (**45**) (Figure 7) were isolated by Luesch et al. from the filamentous marine cyanobacterium *Lyngbya majuscula* found in Piti Bomb Holes, Guam [42]. These are cyclodepsipeptides that have been found to have moderate antimycobacterial activity compared to the standard drug streptomycin, as well as stimulate elastase activity. When tested at 25 μg, the diameter of the zone of growth inhibition for *Mycobacterium tuberculosis* strains ATTC 25177 and ATTC 35818 ranged from 9 to 15 mm for these cyclic depsipeptides. Peng et al. conducted a total synthesis of pitipeptolide A [133], specifically taking interest in the 2,2-dimethyl-3-hydroxyoctynoic acid (Dhoya) unit of the structure, as it is unique to *Lyngbya majuscula*. In addition, pitipeptolide A was found to be a deterrent to urchins, two species of amphipods, and small herbivorous crabs, whereas it did not deter feeding by the sea hare *Stylocheilus striatus*, which readily consumes cyanobacteria [43].

From the same location, additional filamentous marine cyanobacterial samples were collected and further pitipeptolide analogs, namely pitipeptolides C to F, were isolated [44]. Only pitipeptolide F (**46**) (Figure 7) was found to be the most potent in the disk diffusion assay against *Mycobacterium tuberculosis*. Based on the experiments conducted, it was concluded that *N*-methylation in the Phe unit is crucial for antibacterial activity, the π system in the fatty acid unit is not key for antibacterial activity, and a decrease in the hydrophobicity of certain units in the compound could increase the antibacterial potency. Based on the structure–activity relationships established, further modifications and optimization to the structures of the compounds could potentially increase their antibacterial activity.

#### 3.1.10. Pitiprolamide

Pitiprolamide (**47**) (Figure 7), a proline-rich dolastatin 16 analogue, was discovered by Montaser et al. from the filamentous marine cyanobacterium *Lyngbya majuscula,* collected in Piti Bomb Holes, Guam [45]. Compared to dolastatin 16, which has two proline units, pitiprolamide has four proline units. Pitiprolamide was found to have weak antibacterial activities against *Mycobacterium tuberculosis,* starting at 50 µg in a disk diffusion assay, and *Bacillus cereus,* starting at 1 µM in a microtiter plate-based assay, with an approximate IC_50_ value of 70 µM. No antibacterial activities were found against either *Staphylococcus aureus* or *Pseudomonas aeruginosa*.

#### 3.1.11. Hormothamnins

Hormothamnins are a series of cyclic peptides isolated from the filamentous marine cyanobacterium *Hormothamnion enteromorphoides*, collected at Playa de Luquillo, Puerto Rico [46,47]. The total structure of hormothamnin A (**48**) (Figure 7) was determined later by the same group, concluding that it is a cyclic undecapeptide [47]. Hormothamnin A was tested against the Gram-positive bacterium *Bacillus subtilis* and the Gram-negative bacterium *Pseudomonas aeruginosa,* and it was found to be weakly antibacterial against these two strains. Hormothamnin A was also found to be antimicrobial against two human pathogenic microorganisms—*Bacillus subtilis* and *Candida albicans*—through disk diffusion assays, while other hormothamnins, such as hormothamnins A′, C/D, G, G′, G″, J, and K, displayed both antibacterial and antifungal activities [46]. These peptides were also hypothesized to function as deterrents against predators such as fish, zooplankton, and mollusks.

#### 3.1.12. Cyanobacterial Molecules Against Foodborne Pathogens: Antillatoxin B, Laxaphycins, and Malyngamides

**Figure 8:**
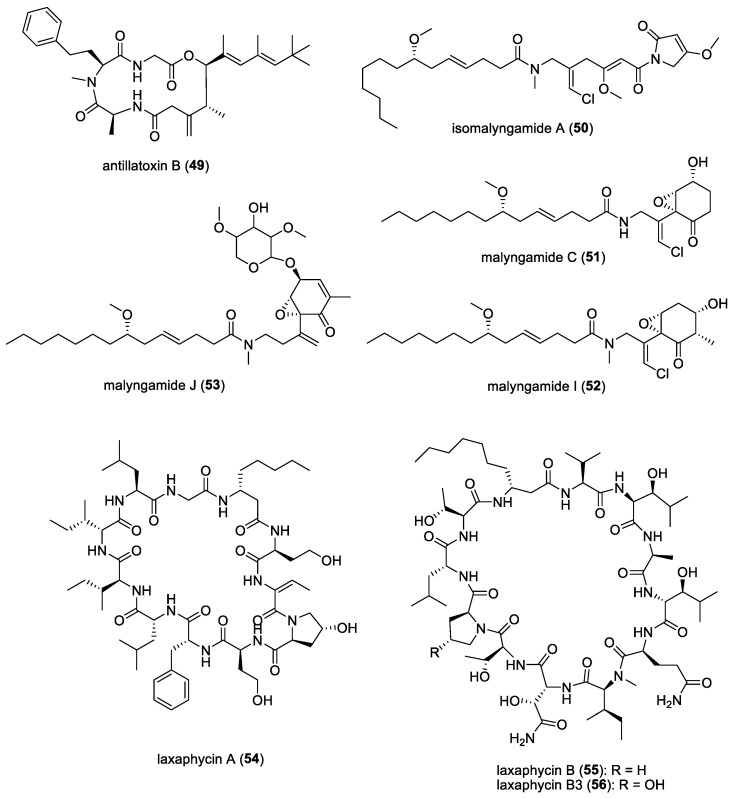
Marine cyanobacterial molecules active against foodborne pathogens.

Food contamination can lead to serious illness [134], which makes the study of foodborne pathogens crucial as well. Several cyanobacterial compounds, namely antillatoxin B (**49**), isomalyngamide A (**50**), malyngamides C (**51**), I (**52**), and J (**53**), as well as laxaphycins A (**54**), B (**55**), and B3 (**56**) (Figure 8), were investigated against five foodborne pathogens in a study conducted by Dussault et al. [135]. These compounds were found to have antibacterial activity against Gram-positive bacterial strains *Listeria monocytogenes*, *Bacillus cereus,* and *Staphylococcus aureus* at low concentrations which were below 500 µg/mL. Interesting compounds to note are isomalyngamide A and malyngamide J, which exhibited antibacterial activity against *Bacillus cereus* at 7.8 µg/mL and 63 µg/mL, respectively. Antillatoxin B and malyngamide C both exhibited antibacterial activity against *Bacillus cereus* at 130 µg/mL, while laxaphycin A inhibited *Staphylococcus aureus* at 125 µg/mL. The rest exhibited antibacterial activity ranging from 250 to 500 µg/mL against the tested bacterial strains.

### 3.2. Quorum Sensing Modulators

#### 3.2.1. Fatty Acids

Marine cyanobacterial-derived fatty acids have been tested as QS and biofilm inhibitors against pathogenic bacteria, such as *Pseudomonas aeruginosa*. In one study, palmitic and oleic acids present in the intracellular methanolic extract of *Oscillatoria subuliformis* were postulated as active compounds against *Pseudomonas aeruginosa*. It was found that the methanolic extract inhibited biofilm (56%), extracellular polymeric substance (40%), cell surface hydrophobicity (56%), pyocyanin (27%), elastase activity, and swarming motility in *P. aeruginosa* [136]. The results revealed that oleic and palmitic acid could be an effective attenuator of *P. aeruginosa* pathogenesis. In another study, palmitic acid from the marine cyanobacterium *Synechococcus elongatus* was investigated for the inhibition of QS-regulated biofilm formation in aquatic bacterial pathogens, including several *Vibrios* sp. It was found that palmitic acid showed significant inhibition of biofilm formation at 100 µg/mL, without interfering with its planktonic growth [137,138]. Further investigations in this study confirm that palmitic acid effectively interferes with the initial adhesion stages of biofilm formation.

#### 3.2.2. Lyngbic Acid

Black band disease (BBD) is a cyanobacteria-dominated polymicrobial disease of corals, and it contains diverse populations of heterotrophic bacteria [139]. It is known to contribute to the degradation of coral reef systems, including those found in the Caribbean, the Indo-Pacific, and the Red Sea. BBD is visually detected by the formation of a dark purple mat caused predominantly by filamentous marine cyanobacteria such as *Roseofilum reptotaenium,* as reported by Casamatta et al. [140]. It has also been suggested that BBD cyanobacteria are involved in structuring the complex polymicrobial BBD microbial community through the production of antimicrobial compounds [139].

In a study by Meyer and co-workers, the cyanobacteria-modified fatty acid lyngbic acid (**41**) (Figure 6) was found to be in abundance within the BBD consortium [41]. It was reported to be a strong QS inhibitor using *Vibrio harveyi* QS reporters. In addition, assays were conducted to confirm the fact that lyngbic acid interferes with CqsS-mediated QS in *Vibrio harveyi* by being the competitive inhibitor of the CAI-1 receptor. Further investigations in situ were conducted with native coral *Vibrio* sp., which was found to possess CAI-1/CqsS-mediated QS. From these experiments, lyngbic acid was indeed found to be a bioluminescence inhibitor of coral vibrios.

#### 3.2.3. Lyngbyoic Acid

**Figure 9:**
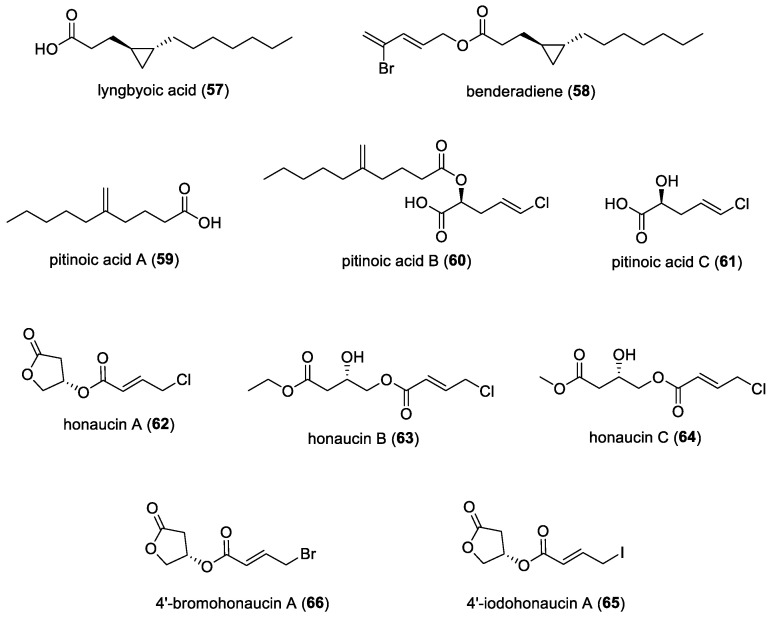
Quorum sensing inhibitors from marine cyanobacteria and synthetic analogs.

A major cyclopropane-containing secondary metabolite of the filamentous marine cyanobacterium *Lyngbya* cf. *majuscula*, lyngbyoic acid (**57**) (Figure 9) was isolated from samples collected in the Indian River Lagoon and Dry Tortugas National Park, Florida [50]. Lyngbyoic acid was found to be effective against one of the acyl homoserine lactone (AHL) receptors—LasR—out of the four AHL receptors tested, including LuxR, AhyR, and TraR. In this study, wild-type *Pseudomonas aeruginosa* was treated with lyngbyoic acid and found to have reduced pigment and elastase production, supported by the reduced expression of the genes required for the biosynthesis of pigment pyocyanin and elastase LasB. Lyngbyoic acid was considered a “tagged” fatty acid, as cyclopropane allowed the compound to persist in producing cyanobacterium and target organisms through the avoidance of metabolism via β-oxidation.

Further investigations show that the AHL-binding site of LasR was not essential to the inhibitory effect of lyngbyoic acid, which suggested that the compound had a dual mechanism, acting both through the AHL-binding site and independently of it. An analysis of global gene expression also showed that lyngbyoic acid down-regulated the majority of genes that had been previously identified as being controlled by QS. Lyngbyoic acid was also tested against gfp-tagged *Pseudomonas aeruginosa* and found to show a significant decrease in biovolume upon treatments with concentrations above 10 µM of lyngbyoic acid [51]. However, no effect was seen on preformed biofilms.

#### 3.2.4. Benderadiene

Benderadiene (**58**) (Figure 9), another cyclopropane-containing molecule, was isolated from the filamentous marine cyanobacterium *Lyngbya majuscula*, found in Singapore [51]. Dose-dependent QS-inhibitory assays were conducted against *Pseudomonas aeruginosa* PAO1 *lasB-gfp* and *rhlA-gfp*, and this molecule was found to have some activity, with IC_50_ values of 89.9 µM and 80.3 µM, respectively. Molecular docking experiments were also conducted to further support bioassay activities, along with lyngbyoic acid, and it was found that, indeed, there was some binding affinity to lasR, similar to the native autoinducer *N*-3-oxo-dodecanoyl-L-homoserine lactone.

#### 3.2.5. Pitinoic Acids

Bioactive pitinoic acids A (**59**)–C (**61**) (Figure 9) were isolated from a Guamanian marine cyanobacterial strain that was morphologically similar to the *Lyngbya* sp., collected from a channel at the north end of Piti Bay, Guam [52]. Pitinoic acid A was found to inhibit QS in *Pseudomonas aeruginosa*, while pitinoic acid B prevented the induction of pro-inflammatory cytokine expression in LPS-induced THP-1 macrophages. Pitinoic acid C was found to maintain the anti-inflammatory activity exhibited by pitinoic acid B. Pitinoic acid B was then concluded to be a naturally occurring prodrug to pitinoic acids A and C. This enables a dual biological activity against the QS of *Pseudomonas aeruginosa* and its inflammatory activity. Pitinoic acid A was found in an abundance of about 0.3% the marine cyanobacteria’s dry weight, which indicated its significance in ecological functions. Moreover, a common biological function of cyanobacterial-derived modified fatty acids, such as lyngbyoic acid, is their interference with the bacterial QS system, which was also proven in this study. Structural similarities between pitinoic acid A and lyngbyoic acid led to the conclusion that they may have similar QS-inhibitory activity.

#### 3.2.6. Malyngolide

In addition to its antibiotic activities, studies by Dobretsov and co-workers reported that malyngolide (**22**) (Figure 4) can also interfere with QS circuitry [27] using *N*-acyl homoserine lactone reporters based on the LasR receptor of *Pseudomonas aeruginosa*. It was found to exhibit inhibitory activities in the range of 3.57 µM to 57 µM, with EC_50_ value of 12.2 µM, without disrupting bacterial growth. It was also found to inhibit elastase production by *Pseudomonas aeruginosa* PAO1 with an EC_50_ value of 10.6 µM. It has also been established in this study that malyngolide is among those secondary metabolites that were not only produced but also released by the cyanobacterium into its surroundings. With these results, it was suggested that malyngolide plays a role in the interactions of heterotrophic bacteria which are associated with the marine cyanobacterium *Lyngbya majuscula*.

In an ecological study by Engene et al., malyngolide was found to keep the newly identified marine cyanobacterium genus *Dapis pleousa* clean from associated microorganisms, as it interferes with the QS systems of Gram-negative bacteria [28]. Due to the unique production of malyngolide from this species, malyngolide was then deemed to be a promising chemotaxonomic marker of *Dapis pleousa*. In another ecological study, malyngolide was among the secondary metabolites produced by cyanobacteria found in the Indian River Lagoon. From this study, it was concluded that malyngolides were able to inhibit the growth of marine fungi *Dendryphiella salina* and *Lindra thalassiae*, which further supports the notion that malyngolides function as a chemical defense, contributing to the persistence of these cyanobacterial blooms [29].

#### 3.2.7. Honaucins

Honaucins A (**62**)–C (**64**) (Figure 9) were isolated from the marine cyanobacterium *Leptolyngbya crossbyana,* found on the Hawaiian coast [53]. It was reported that honaucins A to C were QS inhibitors to the bioluminescence of *Vibrio harveyi* BB120 and *Escherichia coli* JB525. In addition, they were found to inhibit lipopolysaccharide-stimulated nitric oxide production and repress the expression of the pro-inflammatory cytokines in murine macrophages. The above study further investigated the structure–activity relationship of honaucin analogs against QS and inflammatory activities. Through these bioassays, it was found that the key structural feature that is important in the inhibition of QS and inflammation is the halogen attached to the 4′ position of the crotonic acid subunit. Two analogs, 4′-iodohonaucin A (**65**) and 4′-bromohonaucin A (**66**) (Figure 9), were found to be more effective as anti-inflammatory compounds and exhibited better inhibitory effects on QS activities compared to natural honaucins. The 4′-bromohonaucin A was found to be more stable as well, making it a potential lead for the further development of drugs with a dual effect against QS and inflammation.

#### 3.2.8. Tumonoic Acids

**Figure 10:**
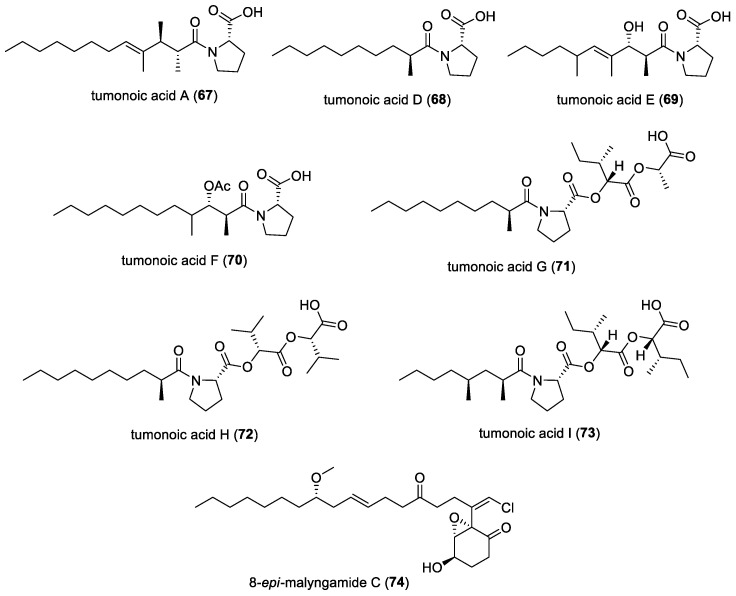
Quorum sensing inhibitors from marine cyanobacteria.

Tumonoic acids A (**67**) and D (**68**)–I (**73**) (Figure 10) were isolated from the marine cyanobacterium *Blennothrix cantharidosmum,* found near the Duke of York Island, Papua New Guinea [54]. These tumonoic acids were the first natural products to be isolated from this genus of marine cyanobacteria and are acyl proline derivatives. Tumonoic acid I was found to display moderate activity as an antimalarial compound, with an IC_50_ of 2 µM, while tumonoic acids E-H were found to inhibit QS systems against a wild-type strain of *Vibrio harveyi*, with tumonoic acid F being the most active, with an IC_50_ value of 62 µM.

#### 3.2.9. Malyngamide C and 8-*epi*-malyngamide C

From a sample of the marine cyanobacterium *Lyngbya majuscula*, collected near Bush Key, Florida, a new stereoisomer of malyngamide C, 8-*epi*-malyngamide C (**74**) (Figure 10), was isolated [48]. The same study also isolated malyngamide C (**51**) (Figure 8) from the sea hare *Stylocheilus longicauda*. Both malyngamide C and 8-*epi*-malyngamide C were found to be cytotoxic to HT29 colon cancer cells and able to inhibit the QS pathway in an LasR-based reporter gene assay without inhibiting bacterial growth, similar to tumonoic acid F.

#### 3.2.10. Doscadenamides

**Figure 11:**
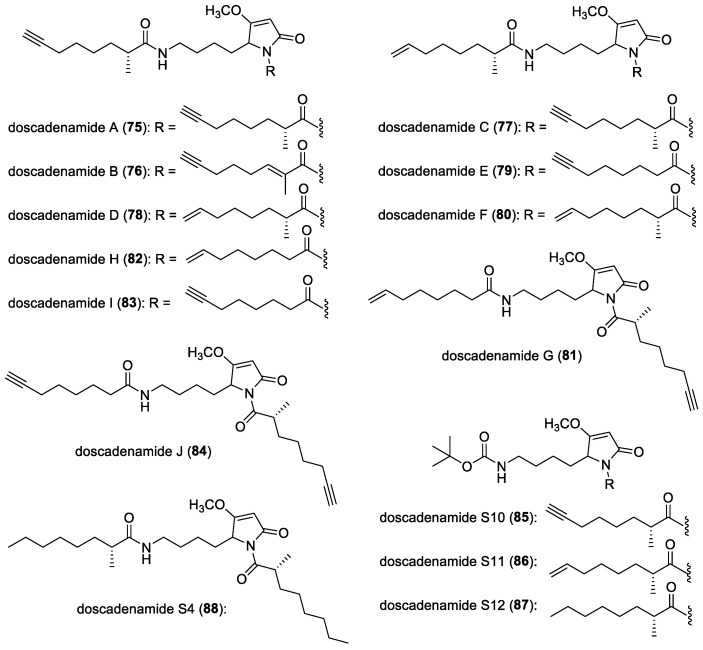
Structures of doscadenamides and synthetic analogs.

A collection of the marine cyanobacterium *Moorena bouillonii*, collected in Fingers Reef, Guam, led to the isolation of doscadenamide A (**75**) (Figure 11), which was found to have similarities with a QS signaling molecule, 3-oxo-C12 HSL, as they both have a five-membered ring core and long alkyl side chains [141]. In addition, a total synthesis of doscadenamide A was developed, and its absolute configuration was confirmed by comparing the isolated natural product with its synthetic diastereomers. It was found that doscadenamide A was able to activate the QS via the AHL-binding site using a reporter plasmid, pSB1075,28, which encodes LasR and has a light-producing luxCDABE cassette, expressed in *E. coli*. Furthermore, the quorum sensing-activating activity of doscadenamide A was verified in wild-type *P. aeruginosa*. This finding suggested that doscadenamide A has the potential to serve as a novel template for developing QS superagonists with new structural frameworks, enabling the further exploration of these activators as therapeutic agents or chemical tools.

Further investigation of the cyanobacterial samples that provided doscadenamide A led to the discovery of new bifunctional analogs, including doscadenamides B (**76**)–J (**84**) (Figure 11) [142]. The structures of doscadenamides B–J were confirmed through total synthesis, and a focused library with varying acylation and unsaturation patterns was synthesized. Structure–activity relationships were explored in various Gram-negative bacteria, including *P. aeruginosa* and *Vibrio harveyi*, and this revealed that the pyrrolinone-*N* acyl chain is essential for full agonist activity, while the second acyl chain can be dispensable or even lead to antagonist activity, depending on the bacterial system. Given that homoserine lactone (HSL)-based QS activators have demonstrated synergy with TRAIL in inducing apoptosis in cancer cells, selected doscadenamides were screened in eukaryotic systems. The most potent QS agonists, doscadenamides S10 (**85**)–S12 (**87**) (Figure 11), along with doscadenamides F (**80**) and S4 (**88**) (Figure 11), which have partial or full saturation of the acyl side chains, showed significant synergistic effects with TRAIL in triple-negative MDA-MB-231 breast cancer cells. The doscadenamide scaffold offers a non-HSL template for combination therapies involving TRAIL pathway activators.

#### 3.2.11. Trikoveramides

**Figure 12:**
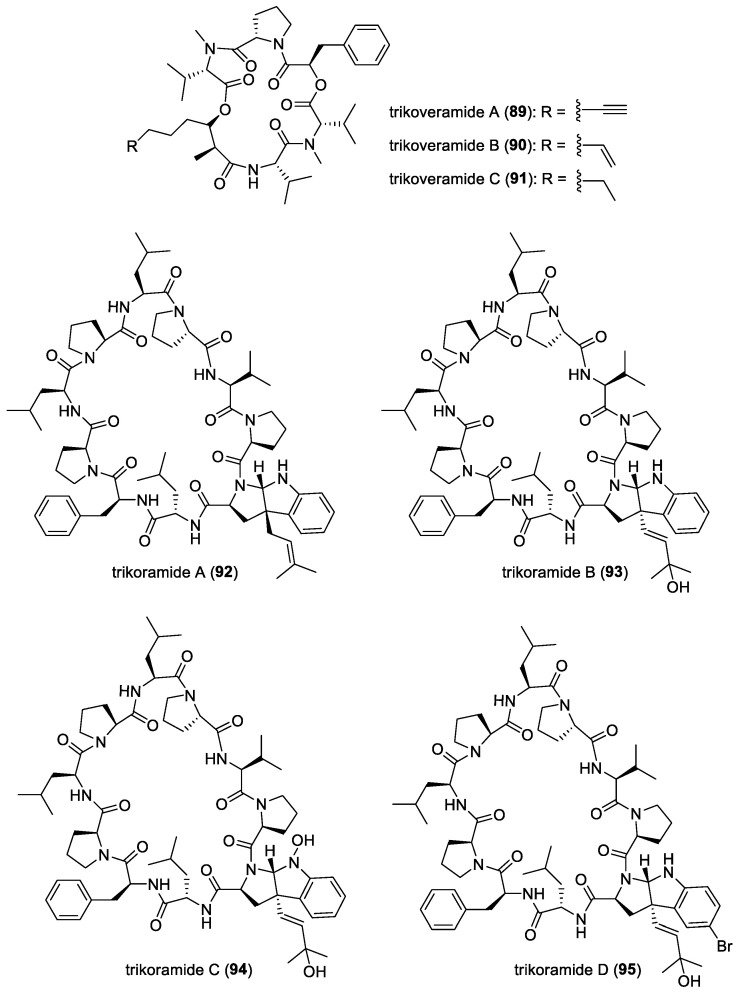
Structures of trikoveramides and trikoramides.

The cyclic depsipeptides trikoveramides A (**89**)–C (**91**) (Figure 12) were isolated from the marine cyanobacterium *Symploca hydnoides*, collected from Bintan [55]. These compounds were tested against MOLT-4 human leukemia cells and were found to be cytotoxic, with IC_50_ values of 48.8 μM, 9.3 μM, and 35.6 μM, respectively. They were also found to have moderate QS-inhibitory activity against *Pseudomonas aeruginosa lasB-gfp* and *rhlA-gfp* bioreporter strains [55].

#### 3.2.12. Trikoramides

Trikoramides A (**92**)–D (**95**) (Figure 12) were isolated from the marine cyanobacterium *Symploca hydnoides*, collected from Bintan. These compounds are C-prenylated cyclotryptophan-containing cyanobactins. Trikoramides A, B, and D exhibited cytotoxicity against the MOLT-4 leukemia cell line, with IC_50_ values of 4.8 μM, 5.2 μM, and 4.7 μM, respectively, and trikoramide B exhibited QS-inhibitory activities against PAO1 *lasB-gfp* and *rhlA-gfp*, with IC_50_ values of 19.6 μM and 7.3 μM, respectively [56,143].

## 4. Antifungals

Cyanobacterial metabolites are recognized for their unique structural characteristics, which contribute to significant bioactivity and stability. However, only a small fraction of these compounds have been explored for antifungal properties [144]. Among those studied, many exhibit potent inhibitory effects, surpassing commercial antifungals in their ability to combat multidrug-resistant strains. These metabolites span various chemical classes, such as peptides, fatty acids, alkaloids, polyketides, and macrolides. Additionally, they can target a wide range of cellular components. The following section provides details on antifungals isolated from marine cyanobacteria.

### 4.1. Majusculoic Acid

**Figure 13:**
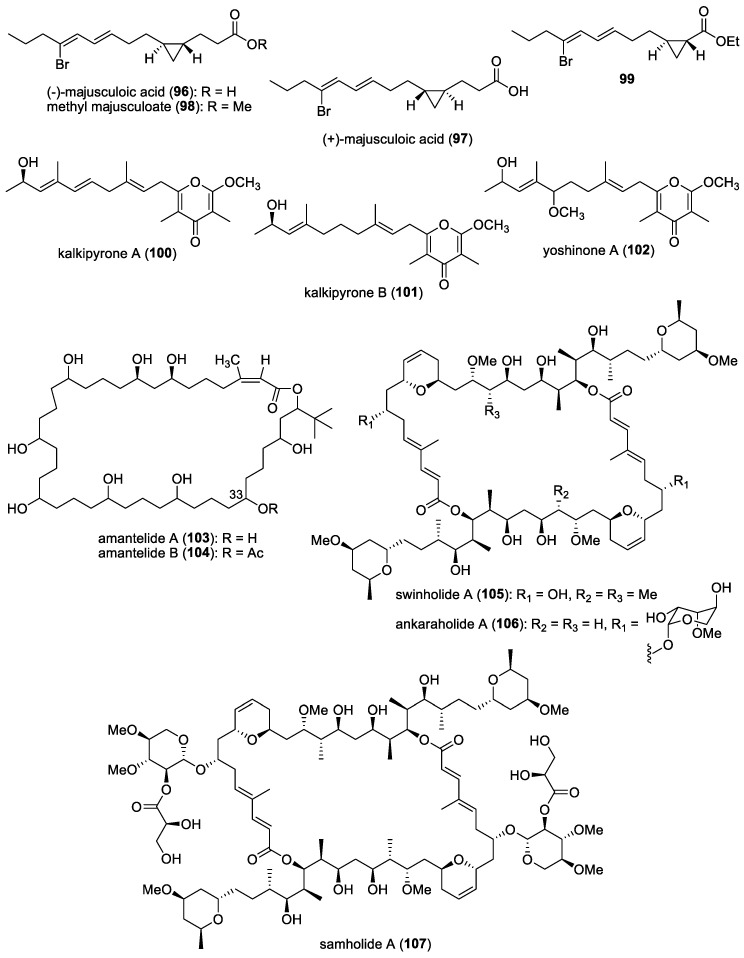
Antifungal marine cyanobacterial and synthetic molecules.

A novel antifungal cyclopropane-containing molecule, majusculoic acid (**96**) (Figure 13), was discovered from a cyanobacterial mat obtained from a shallow inlet, in Sweetings Cay, Bahamas [57]. Majusculoic acid was found to exhibit antifungal properties against *Candida albicans* ATCC 14503 and *C. glabrata*, with MIC values of 8 μM and 19.3 μM, respectively. The first total synthesis of (+)-majusculoic acid (**97**) (Figure 13), an enantiomer of the natural majusculoic acid, was carried out in 13 steps, involving ring-closing metathesis dimerization, conformationally controlled cyclopropanation, dedimerization, and bromoolefination [145]. The subsequent total synthesis of (−)-majusculoic acid (**96**) and its derivatives was achieved by Xiao and co-workers, featuring the application of the conformational controlled establishment of trans-cyclopropane and stereochemical-controlled bromo-olefination or olefination by Horner–Wadsworth–Emmons reaction [146]. In addition, synthetic (−)-majusculoic acid, methyl majusculoate (**98**), and ethyl-(1*R*,2*R*)-2-((3*E*,5*Z*)-6-bromonona-3,5-dien-1-yl)cyclopropane-1-carboxylate (**99**) (Figure 13) exhibited significant inhibition of nitric oxide production in lipopolysaccharide (LPS)-induced mouse macrophage RAW264.7, suggesting their potential application as anti-inflammatory agents.

### 4.2. Tanikolide

Tanikolide (**17**) (Figure 4), structurally related to malyngolide, was isolated in 1999 from *L. majuscula* collected from shallow water on Tanikeli Island, Madagascar [25]. It exhibited antifungal activity against *Candida albicans,* with a 13 mm diameter zone of inhibition when tested at 100 µg/disk using the paper disk–agar plate method. The molecule also showed molluscicidal activity against *Biomphalaria glabrata*, with an LD_50_ of 9.0 μg/mL. The related compound, malyngolide, having an opposite configuration at C-5 to tanikolide, was not active against *C. albicans*. Since the discovery of tanikolide, many synthetic efforts regarding the total synthesis of tanikolide and its derivatives have been reported [147,148,149,150,151,152,153,154,155,156,157].

### 4.3. Kalkipyrones A and B

Two γ-pyrone-containing compounds, kalkipyrones A (**100**) and B (**101**), along with a known related molecule, yoshinone A (**102**) (Figure 13), were isolated from field-collections of the cyanobacteria *Leptolyngbya* sp., from Fagasa Bay, American Samoa, and cf. *Schizothrix* sp., Panama, respectively [58]. Kalkipyrone A was also reported from an assemblage of the marine cyanobacteria *Lyngbya majuscula* and *Tolypothrix* sp., collected in Playa Kalki, Curacao [59]. Only kalkipyrones A and B showed moderate toxicity against *Saccharomyces cerevisiae* ABC16-Monster strain, with IC_50_ values of 14.6 and 13.4 μM, respectively. Yoshinone A was also found to have low toxicity to the *S. cerevisiae* strain, with an IC_50_ of 63.8 μM.

### 4.4. Amantelides

Two cytotoxic polyhydroxylated macrolides, amantelides A (**103**) and B (**104**) (Figure 13), were isolated from a gray cyanobacterium obtained from Two Lover’s Point (Puntan dos Amantes), Tumon Bay, Guam [60]. These samples are likely related to the genus *Okeania,* but molecular vouchers are lacking. Amantelides are characterized by a 40-membered macrolactone ring, having a 1,3-diol and contiguous 1,5-diol units as well as a *tert*-butyl substituent. A series of bioactivity evaluations revealed the broad-spectrum property of amantelide A against eukaryotic and prokaryotic cells. In addition to its cytotoxic activity, amantelide A completely inhibited the growth of three marine fungi, including *Dendryphiella salina*, *Lindra thalassiae,* and *Fusarium* sp., when tested at its estimated natural concentration of 625 μg/mL. This observation suggests the ecological role of amantelide A as a defensive molecule against these marine fungal pathogens. At a 10-fold-lower concentration of 62.5 μg/mL, amantelide A continues to completely inhibit the growth of *L. thalassiae* and *Fusarium* sp. In comparison, the known amphotericin B completely inhibited *D. salina* growth but had a minimal effect on the growth of *L. thalassiae* and *Fusarium* sp.

The possible mode of action of amantelide A on the cell membrane was hypothesized based on the biological activities of related polyhydroxylated compounds. A subsequent study by Elsadek and co-workers showed amantelide A to exert its antifungal activity by binding to ergosterol-containing membranes, followed by pore formation, similar to the activity of other polyene antifungals, including nystatin [158]. In vitro binding assays revealed the significant binding of amantelide A to 1-palmitoyl-2-oleoyl-sn-glycero-3-phosphatidylcholine-based liposomes. Binding was also shown to markedly increase after the inclusion of 20 mol% ergosterol in the liposome. Moreover, increased affinity was observed with membranes containing cholesterol, suggesting that amantelide A’s cytotoxicity to mammalian cells is due to its affinity to cholesterol-containing membranes. Upon the binding of amantelide A to the membrane, a pore or a lesion with a diameter of 2.0–3.8 nm could be formed, similar to that of amphidinol 3. It has been suggested that the hydroxy group at C-33 of amantelide A could be involved in this unique membrane binding, since the C-33 acetoxy derivative amantelide B did not show sterol-dependent membrane affinity and biological activities.

### 4.5. Swinholide-Related Molecules

Swinholides are a unique family of macrolide natural products characterized by a dimeric 44-membered or larger lactone ring. The first member of this family, swinholide A (**105**) (Figure 13), was isolated in 1985 by Kashman and Carmeli from the marine sponge *Theonella swinhoei* [61]. These swinholide-like compounds, including tolytoxin and scytophycins, had already been identified in terrestrial cyanobacteria by Moore’s research group [159]. Tolytoxin exhibited potent cytotoxic and fungicidal properties and was later found to share structural similarities with a monomeric portion of swinholide A. In addition, scytophycins, isolated from the cultured terrestrial cyanobacterium *Scytonema pseudohofmanni*, demonstrated strong cytotoxic and broad-spectrum antifungal activities. The molecular target of swinholides in mammalian cells is actin, with some compounds exhibiting nanomolar potency at the same binding sites on F-actin and G-actin [160].

In 2005, two new glycosylated swinholides, ankaraholides A (**106**) and B (Figure 13), were identified from a Madagascan (at Nosy Mitso-ankaraha Island) cyanobacterium belonging to the genus *Geitlerinema*, along with swinholide A from a Fijian cyanobacterium, *Symploca* cf. sp. [161]. Additionally, nine new derivatives, samholides A (**107**)–I (Figure 13), were obtained through a bioassay-guided isolation approach combined with MS2-based molecular networking from a field collection of the cyanobacterium cf. *Phormidium* sp. in American Samoa [162]. These marine cyanobacterial-derived swinholide-related compounds have not been tested for their antifungal activities.

### 4.6. Dolastatin 10

**Figure 14:**
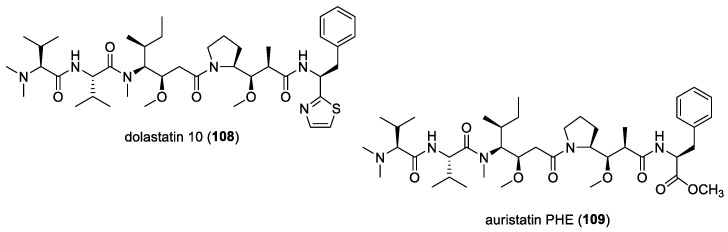
Antifungal dolastatin 10 and auristatin PHE.

Originally isolated from the Indian Ocean sea hare *Dolabella auricularia*, dolastatin 10 (**108**) (Figure 14) is a linear peptide having remarkable cytostatic and antineoplastic properties [163]. This molecule is now known to be produced by marine cyanobacteria due to its re-isolation from *Symploca* sp. VP642 collected from Palau [164]. In addition to cytotoxicity, the antifungal activity of dolastatin 10 and its derivative, auristatin PHE (**109**) (Figure 14), against several yeasts and filamentous fungi, with selective activity against *Cryptococcus neoformans*, was reported [165,166]. Auristatin PHE was found to exhibit extremely low MICs for *C. neoformans,* and its antifungal activity was largely not affected by pH changes and had enhanced activity in the presence of human serum. Subsequent studies showed auristatin PHE to possess specificity for *C. neoformans* and several species of *Trichosporon* based on broth microdilution assays [161]. Moreover, the post-antifungal effect (PAFE) for this derivative was detectable after 45 min of exposure, and its effect plateaued after 1 h of exposure, having a PAFE of approximately 6.5 h at four or eight times the auristatin PHE MIC. Furthermore, human serum significantly prolonged the PAFE of auristatin PHE at eight times the MIC. It was also found that auristatin PHE arrested *C. neoformans* in the budding stage, possibly due to a tubulin-inhibitory activity [167]. Its cell-arresting effects were further studied, and researchers found that the molecule disruption of the microtubules was accompanied by the blockage of nuclear migration and nuclear and cellular division, which led to cells arrested in a uninucleate, large-budded stage [168]. Investigation of the effects of auristatin PHE on differential gene expression in *C. neoformans* revealed that fungal cells treated with 1.5 times the MIC of auristatin PHE for 90 min showed 29 transcript expression differences between the control and drug-treated populations. Interestingly, the genes found to be differentially expressed were those encoding proteins related to transport, cell cycle regulation, signal transduction, cell stress, DNA repair, nucleotide metabolism, and capsule production [169].

### 4.7. Majusculamide C and Related Compounds

**Figure 15:**
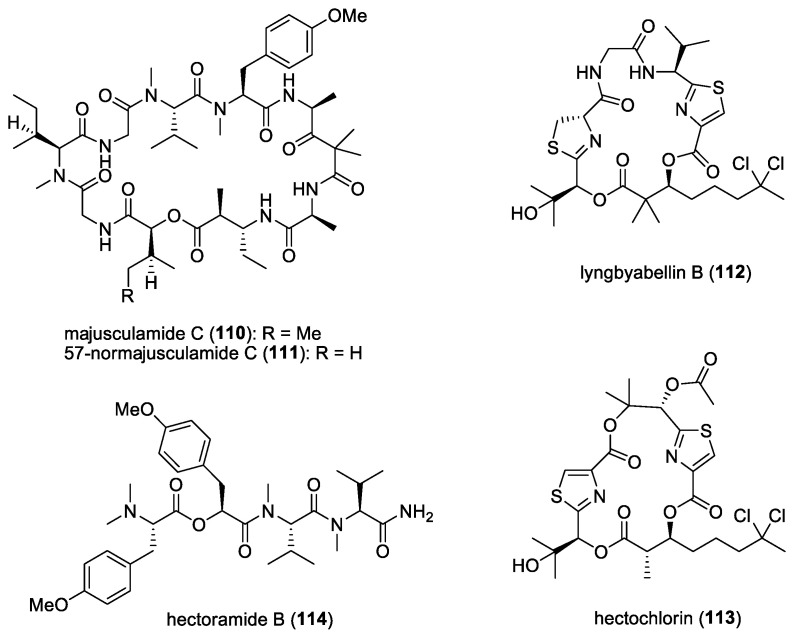
Antifungal cyclic depsipeptides and hectoramide B.

From a deepwater variety of *L*. *majuscula* collected from the lagoon of the Enewetak Atoll, Marshall Islands, a cyclic depsipeptide, majusculamide C (**110**) (Figure 15), was found to significantly inhibit the growth of fungal plant pathogens, including *Phytophthora infestans*, *Plasmopora viticola*, and *Rhizoctonia solani*, the causative organisms of tomato late blight, grape downy mildew, and Rhizoctonia damping-off, respectively [62]. A related molecule, 57-normajusculamide C (**111**) (Figure 15), was later found to display antimycotic properties against *Saccharomyces pastorianus* [63].

### 4.8. Lyngbyabellin B and Hectochlorin

At least two marine cyanobacterial thiazole-containing cyclic depsipeptides have been reported to possess antifungal activities. Lyngbyabellin B (**112**) (Figure 15), isolated from *L. majuscula* collected from the Dry Tortugas National Park, Florida, showed antifungal activity against *C. albicans* (ATCC 14053), with a 10.5 mm zone of inhibition at 100 μg/disk using a disk diffusion assay [64]. The Lyngbyabellin family is a fairly large class of molecules consisting of at least 17 related compounds. However, only lyngbyabellin B has been evaluated for its antifungal activity. Given its structural similarities, other members of the Lyngbyabellin class should exhibit antifungal properties. Another antifungal compound, hectochlorin (**113**) (Figure 15), isolated from *L. majuscula* found in Hector Bay, Jamaica, and Boca del Drago Beach, Panama, showed a 16 mm zone of inhibition at 100 μg/disk and an 11 mm zone of inhibition at 10 μg/disk against *C. albicans* (ATCC 14053) [65]. Since their discovery, several efforts have been reported on their total synthesis [170,171]. One of these synthetic efforts was initiated due to the discovery, by an agrochemical company, that hectochlorin exhibited potent antifungal activity against several crop disease fungi [171].

The hectochlorin biosynthetic gene cluster (*hct*) has also been isolated from *Lyngbya majuscula* [172]. The cluster comprises eight open reading frames (ORFs), spanning 38 kb, with a genetic architecture and domain organization that appears to be colinear with its biosynthetic process. Notably, two peptide synthetase modules contain ketoreductase (KR) domains, which are believed to play a role in synthesizing the 2,3-dihydroxyisovaleric acid (Dhiv) units. Additionally, two cytochrome P450 monooxygenases, located at the downstream end of the cluster, are likely involved in Dhiv formation. A putative halogenase at the beginning of the cluster is predicted to catalyze the formation of 5,5-dichlorohexanoic acid.

The Jamaican marine cyanobacterial strain that produces hectochlorin has now been reclassified as *Moorena producens* JHB and has been continuously cultivated in seawater-BG11 culture medium since its original collection [173]. In addition to antifungal hectochlorins A–D, other bioactive compounds have been isolated, including sodium channel antagonists jamaicamides A−F, cryptomaldamide, and hectoramide A. A recent report on the co-culturing of this strain with *C. albicans* led to the production of a new compound, hectoramide B (**114**) (Figure 15). Although anti-Candida activity was not detected for this newly expressed compound, the authors suggested that hectoramide B could still be important in protecting *M. producens* JHB from possible antagonistic effects by *C. albicans* [173].

### 4.9. Laxaphycins and Lobocyclamides

Laxaphycins are a large family of cyclic lipopeptides, characterized by a rare β-amino fatty acid with a short linear chain of either eight or ten carbons. Laxaphycins were first reported by Moore’s research group in Hawaii in 1992 from the terrestrial cyanobacterium *Anabaena laxa* [174,175]. Five years later, a report by Bonnard and co-workers identified their complete structures, including the absolute stereochemistry of the amino acids constituting the sequence of laxaphycins A (**54**) and B (**55**) [176] (Figure 8). These two cyclic lipopeptides were isolated from an assemblage of the marine cyanobacteria *L. majuscula* and *Anabaena torulosa*, collected in the Moorea Atoll, French Polynesia. The latter marine cyanobacterial species was later identified as the source of laxaphycins upon repeated collections in the Moorea Atoll [49]. Structurally, laxaphycin A has segregated hydrophobic and hydrophilic residues, and laxaphycin B has alternating hydrophobic and hydrophilic residues. Laxaphycin B, a dodecapeptide, was found to have antifungal activity against *C. albicans*, while laxaphycin A, an undecapeptide, was inactive. Interestingly, laxaphycin A potentiates the antifungal activity of laxaphycin B when the two compounds are combined. A subsequent biosynthetic study revealed that the synergistic activity of laxaphycins was linked to their shared biosynthesis mode [177]. The first total synthesis of laxaphycin B was achieved by Boyaud and co-workers using stepwise automated solid-phase peptide synthesis [178].

**Figure 16:**
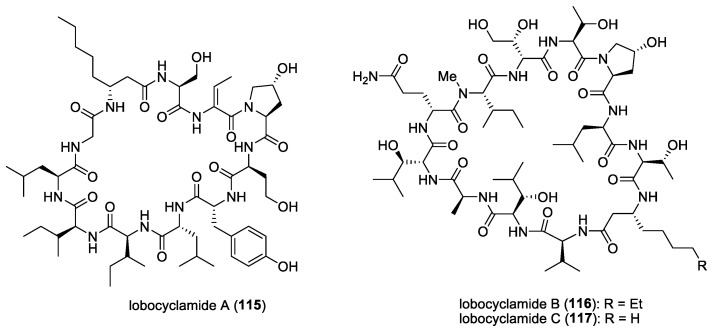
Chemical structures of lobocyclamides.

A series of laxaphycin-related antifungal lipopeptides, lobocyclamides A (**115**)–C (**117**) (Figure 16), were purified from a benthic samples of *Lyngbya confervoides* obtained from Cay Lobos, Bahamas [66]. The absolute configuration of the 3-hydroxyleucine unit in lobocyclamide B was determined through synthesis involving an aldolization reaction [179]. These molecules exhibited modest antifungal activity when tested against fluconazole-resistant fungi *C. albicans* 96–489 and *C. glabrata* in disk diffusion assays on Saboraub agar plates. Interestingly, mixtures of lobocyclamides A and B in a 1:1 ratio showed synergistic antifungal activity with an MIC value of 10–30 μg/mL. Such synergistic activity was also observed for related compounds—laxaphycins A and B.

## 5. Antiparasitics

Parasitic diseases impact millions worldwide, particularly in developing countries where poor sanitary and hygiene conditions lead to high mortality rates [180]. Despite this significant health burden, the therapeutic options for treating these diseases have remained largely unchanged for many years, characterized by a low effectiveness and numerous side effects [181]. Parasitic diseases can be classified into two main categories: those caused by protozoan parasites, including *Plasmodium* (malaria), *Leishmania* (leishmaniasis), *Trypanosoma* (African trypanosomiasis and Chagas disease), and *Toxoplasma gondii* (toxoplasmosis) and those caused by helminths, such as *Schistosoma mansoni* (schistosomiasis) and *Taenia solium* (taeniiasis). The emergence of resistance to both past and current antimalarial drugs underscores the ongoing need for research to stay ahead of the disease [182]. There is a critical need for new drugs, particularly those with novel mechanisms of action. Although progress has been made in reducing the burden of human African trypanosomiasis, there is still an urgent need for new treatments for leishmaniasis and Chagas disease [183]. This section provides up-to-date information regarding the isolation and ongoing studies of selected marine cyanobacterial compounds with notable activity against *Plasmodium falciparum*, *Leishmania* sp., and *Trypanosoma* sp.

### 5.1. Antiplasmodial Molecules

#### 5.1.1. Hierridin B and 2,4-Dimethoxy-6-heptadecyl-phenol (= Hierridin A)

**Figure 17:**
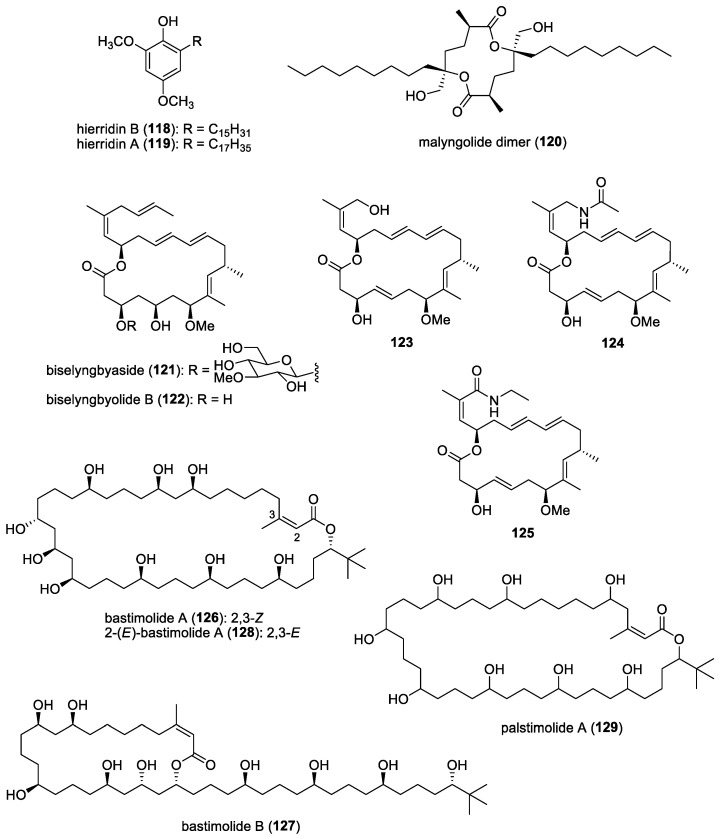
Antiparasitic marine cyanobacterial molecules.

The bioassay-guided fractionation of the CH_2_Cl_2_ extract of the marine cyanobacterium *Phormidium ectocarpi* provided a new natural product, hierridin B (**118**), and the known 2,4-dimethoxy-6-heptadecyl-phenol (**119**) (= hierridin A) (Figure 17) [67]. When tested as a mixture, it exhibited antiplasmodial activity against two strains of *P. falciparum*, namely clones D6 (chloroquine-sensitive) and W2 (chloroquine-resistant), with IC_50_s of 5.2 μg/mL and 3.7 μg/mL, respectively. Due to the structural similarities of hierridin B and 2,4-dimethoxy-6-heptadecyl-phenol to other quinones reported to exhibit in vitro antiplasmodial activity, it has been suggested that these cyanobacterial molecules could act as co-enzyme Q anti-metabolites, causing oxidative stress in protozoans.

#### 5.1.2. Malyngolide Dimer

A dimeric structure of malyngolide, the malyngolide dimer (**120**) (Figure 17), was isolated from the Panamanian marine cyanobacterium *Lyngbya majuscula* in 2010 [68]. The dimer was reported to exhibit moderate in vitro activity against the chloroquine-resistant *Plasmodium falciparum* strain W2, with an IC_50_ of 19 μM. It also showed a similar level of toxicity when tested against the H-460 human lung cell line. The isolation of the malyngolide dimer as well as other dimeric natural products, such as the tanikolide dimer and swinholide, suggested the general biosynthetic capacity of cyanobacteria to dimerize these polyketide-derived molecules.

#### 5.1.3. Biselyngbyaside and Biselyngbyolide B

In 2015, Morita and co-workers showed that cytotoxic biselyngbyaside (**121**) (Figure 17) and its aglycone analogue, biselyngbyolide B (**122**) (Figure 17), inhibited the activities of SERCA1a and 2a and determined the crystal structures of these molecules, binding to SERCA1a [184]. Biselyngbyaside and its natural derivatives were originally reported from the marine cyanobacterium *Lyngbya* sp., collected in Okinawa [185,186]. SERCA-type pumps are important membrane proteins that are involved in various cellular functions and, hence, are conserved in a wide range of organisms, including an SERCA orthologue, PfATO6, found in *P. falciparum* [187]. Previous studies also suggested that PfATP6 plays an important role in malaria disease and may be a potential molecular target of antimalarial drugs [188]. Subsequently, biselyngbyaside was tested for antimalarial activity against chloroquine-resistant K1 and chloroquine-sensitive FCR3 strains and showed IC_50_ values of 3.4 μM and 4.4 μM, respectively [69]. However, biselyngbyolide B showed much weaker antimalarial activities (K1, IC_50_ 24.0 μM; and FCR3, IC_50_ 23.5 μM) compared to biselyngbyaside. In spite of its weaker antimalarial activity, biselyngbyolide B was selected as a structural template for the synthesis of derivatives due to its similar *K*i value to biselyngbyaside and its simpler structure. By modifying the synthetic route for biselyngbyolide B, three analogs of biselyngbyolide B, namely **123** to **125** (Figure 17), with hydrophilic groups on the side chain were synthesized [69]. Unfortunately, these synthetic analogs did not show improvements in antimalarial activity. It has been suggested that other changes, including adding a sugar moiety or a macrolatone ring structure, could be made to improve the antimalarial properties of this group.

#### 5.1.4. Bastimolides and Palstimolide A

Antimalarial bastimolides A (**126**) and B (**127**) (Figure 17) were isolated from a collection of *Okeania hirsuta*, obtained in the vicinity of Isla Bastimentos Park, Panama [189,190]. The former molecule is a 40-membered ring polyhydroxy macrolide, while the latter compound is a 24-membered polyhydroxy macrolide. Both molecules contain a rare tert-butyl terminus. Bastimolide A exhibits highly potent activity against four multidrug-resistant strains of *Plasmodium falciparum*, including TM90-C2A, TM90-C2B, W2, and TM91-C235, with IC_50_ values ranging from 80 to 270 nM. Bastimolide B shows strong antimalarial activity against the CQ-sensitive *P. falciparum* strain HB3, with an IC_50_ of 5.7 μM. The methanolysis of bastimolide A provides an unexpected isomerization product of the C2–C3 double bond, 2-(*E*)-bastimolide A (**128**) (Figure 17), and this synthetic molecule exhibits the greatest potency against the chloroquine-sensitive *P. falciparum* strain HB3, with an IC_50_ of 1.4 μM [190]. Three acetonide synthetic derivatives of bastimolide A have also been evaluated, which revealed that the 9-OH/11-OH groups in bastimolide A have little effect on the antimalarial activity of the molecules in question [190]. Due to their potent antimalarial activity, several attempts at a total synthesis of bastimolides have been reported [191,192,193]. A short enantioselective catalytic synthesis of the key C15−C27 fragment in bastimolide A has also been carried out to facilitate the strategic insertion of halogen atoms, such as fluorine and chlorine atoms, for the possible synthesis of halogenohydrin-containing bastimolide analogs [194].

A related 40-membered macrolide, palstimolide A (**129**) (Figure 17), was isolated from a tropical cyanobacterium, *Leptolyngbya* sp., collected in the Palmyra Atoll, in the Central Pacific Ocean [195]. Palstimolide A had structural similarities to other 40-membered polyhydroxylated macrolides, including bastimolide A, amantelides, and nuiapolide. It displayed potent antimalarial activity against the blood stage of the *P. falciparum* Dd2 strain, with an IC_50_ of 172.5 nM, as well as the intracellular *Leishmania donovani* parasite infecting murine macrophage cells, with an IC_50_ of 4.67 μM.

#### 5.1.5. Dolastatins

**Figure 18:**
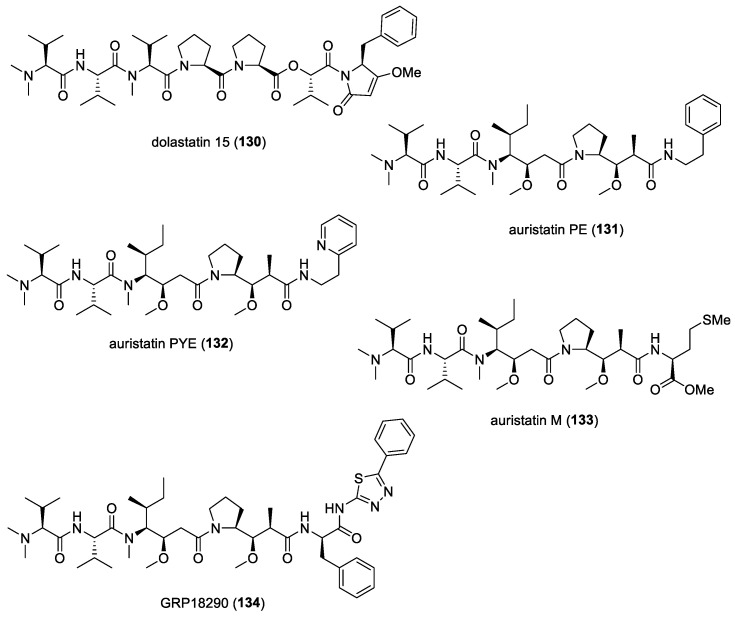
Dolastatin 15 and synthetic dolastatin 10 analogs with antiparasitic activity.

In addition to their cytotoxic properties, dolastatins 10 (**108**) and 15 (**130**) (Figure 18) were revealed to show antimalarial properties against *P. falciparum*. The former peptidic molecule was a more potent inhibitor of the *P. falciparum* FCH5.C2 strain than any other previously described microtubule inhibitor, having an IC_50_ value of 0.1 nM [196]. Dolastatin 15 was found to be less active, with an IC_50_ at 200 nM. A series of ten dolastatin 10 synthetic derivatives were also evaluated for antimalarial activity, and researchers found that auristatin PE (**131**), auristatin PYE (**132**), auristatin M (**133**), GRP18112 [= auristatin PHE (**109**)], and GRP18290 (**134**) (Figure 18) had IC_50_ values ranging from 0.34 to 5.2 nM. Moreover, dolastatin 10 and auristatin PE caused arrested nuclear division and the disassembly of mitotic microtubular structures in plasmodial parasites. These studies showed that molecules binding to the ‘Vinca domain’ of tubulin, such as dolastatin 10, can possess potent antimalarial properties.

#### 5.1.6. Gallinamide A (= Symplostatin 4)

As part of an ongoing initiative to discover novel treatments for neglected parasitic diseases, the Panama International Cooperative Biodiversity Group (ICBG) program has been exploring the antimalarial potential of secondary metabolites from Panamanian marine cyanobacteria. Among the over 60 cyanobacteria strains evaluated in their biological screens, the organic extract of a *Schizothrix* species, obtained from a reef near Piedras Gallinas, Panama, demonstrated significant initial antimalarial activity against the W2 chloroquine-resistant strain of *P. falciparum*. Subsequent bioassay-guided fractionation led to the isolation of a new highly functionalized linear peptide, gallinamide A (**10**) (Figure 2) [13]. This molecule exhibited moderate in vitro activity against *P. falciparum* and *Leishmania donovani,* with IC_50_ values of 8.4 μM and 9.3 μM, respectively.

Based on the total synthesis of gallinamide A, it was confirmed that its structure and stereochemistry were identical to antimalarial symplostatin 4, previously reported by Leusch and co-workers in a Floridian marine cyanobacterium, *Symploca* sp. [118,197,198]. Interestingly, the three synthetic distereoisomers of gallinamide A were found to be active against the 3D7 strain of *P. falciparum*, with IC_50_ values ranging from 36.8 to 104 nM [197]. An important biological feature of gallinamide A is the fact that it did not show hemolytic activity in red blood cells. Subsequently, the molecule’s mode of action for the inhibition of a group of cysteine proteases, known as falcipains, found within the food vacuole of the parasite, was uncovered by Stolze et al. [119]. In fact, all three types of falcipains, namely FP2, FP2′, and FP3, localized within the food vacuole, were inhibited by gallinamide A at low- to mid-nanomolar concentrations.

**Figure 19:**
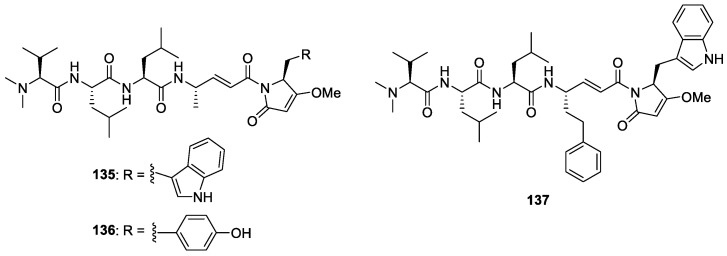
Antiparasitic synthetic gallinamide A analogs.

Several potent new synthetic inhibitors of the food vacuole FPs, including FP2 and FP3, were discovered based on the structure of gallinamide A [199]. The crucial role of the α,β-unsaturated imide moiety in the natural product’s inhibitory activity was initially demonstrated by synthesizing selectively reduced analogs and evaluating several derivatives with a C-terminal amide replacing the imide functionality. By varying the side chain on the pyrrolinone ring, several potent inhibitors of FP-2 and FP-3, such as **135** and **136** (Figure 19), were identified. Additionally, many of these compounds showed strong inhibition of the CQ-sensitive 3D7 strain of *P. falciparum*, with several molecules proving to be as potent as CQ. Notably, these analogs also retained potent activity against the CQ-resistant Dd2 strain of *P. falciparum* and exhibited no significant toxicity to HEK298 cells.

A second-generation library of gallinamide A analogs that retained the key features for inhibitory potency against FPs and *P. falciparum* in vitro was reported by Stoye et al. [200]. They managed to identify compounds with potent in vitro activity against the intravacuolar cysteine proteases FP-2 and FP-3, as well as *P. falciparum* parasite growth. Their efforts provided valuable insights into how structural changes affect antiplasmodial activity and plasma and metabolic stability. Suitable candidates were then evaluated for their efficacy against *P. berghei* malaria infection in mice. One analogue, **137** (Figure 19), successfully cured murine malaria in a Peters 4-day suppressive test in all test group animals at doses of 25 mg/kg and also proved effective in a therapeutic model at 50 mg/kg. Additionally, this compound showed promising activity when administered orally at 100 mg/kg in established infections.

#### 5.1.7. Carmaphycin B

**Figure 20:**
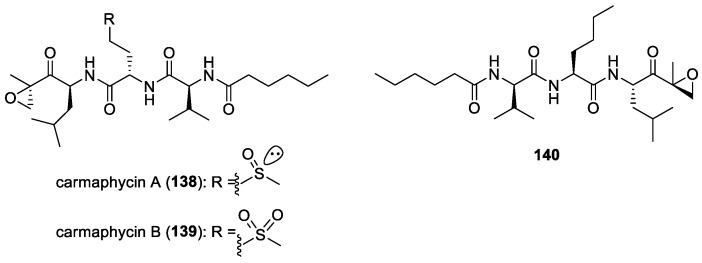
Structures of carmaphycins and synthetic analog.

Carmaphycins A (**138**) and B (**139**) (Figure 20) are modified tripeptides isolated from the cyanobacterium *Symploca* sp. collected off an anchor rope by a snorkeler south of the CARMABI research station in Curacao [70]. Carmaphycins A and B contain an α, β-epoxyketone warhead and have been found to inhibit the β5 subunit of the 20S proteasome of the yeast *Saccharomyces cerevisiae*. In subsequent studies, carmaphycin B was found to exhibit potent activity against the asexual, liver, and sexual stages of the *Plasmodium falciparum* Dd2 strain, with IC_50_ values of 4.1, 61.6, and 160 nM, respectively [201]. New compounds based on carmaphycins have been synthesized, and they have shown enhanced selectivity and activity against the *P. falciparum* 20S proteasome. The proteasome is a multi-subunit enzyme complex responsible for degrading short-lived, abnormal, or damaged proteins in eukaryotic cells. Protozoan parasites, which experience rapid growth and cell division, are particularly vulnerable to proteotoxic stress and, therefore, depend heavily on their protein quality control systems for survival. As such, the proteasome has recently gained attention as a promising drug target [202]. Among the carmaphycin-related synthetic analogs, **140** (Figure 20) displayed a therapeutic window 100 times broader than carmaphycin B. It features substitutions of D-valine for L-valine and norleucine for methionine sulfone. In addition, this synthetic compound retains potent antimalarial activity in cell-based assays against both asexual blood stages and gametocytes, and it significantly inhibits the activity of the isolated *Plasmodium* proteasome in vitro. Moreover, in vitro evolution studies in *S. cerevisiae*, along with biochemical assays and molecular modeling, confirm that its activity results from the specific inhibition of the proteasome’s β5 subunit. Furthermore, carmaphycin B synergizes with artemisinin for the elimination of plasmodial parasites. The co-treatment of wild-type parasites with artemisinin and carmaphycin B or analog **140** resulted in 2-fold and 3-fold sensitization to artemisinin, resulting in an induction of oxidative stress in the parasite, with an increase in oxidized dysfunctional proteins.

#### 5.1.8. Carmabin A, Dragomabin, and Dragonamides A and B

**Figure 21:**
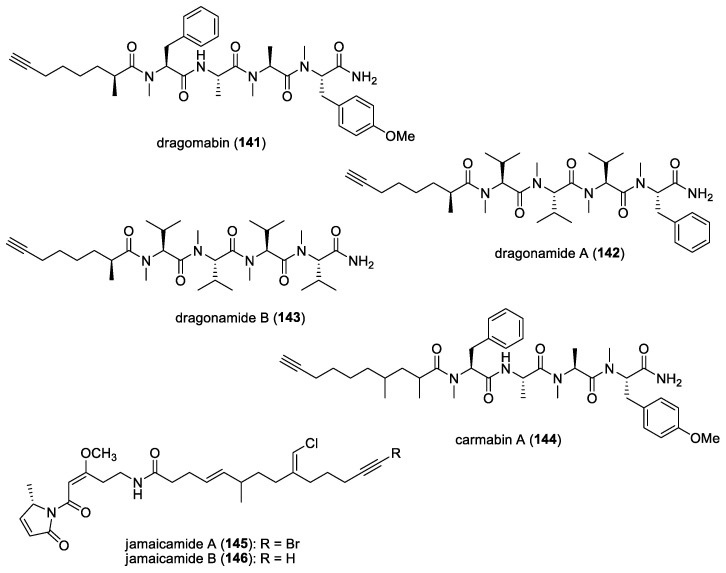
Marine cyanobacterial molecules with antiparasitic activity.

The antimalarial bioassay-guided isolation of extracts prepared from four separate collections of Panamanian *Lyngbya majuscula* in Isla Bastimentos (two separate collections), Bocas del Drago, and Crawl Cay in Bocas del Toro led to the reporting of two new lipopeptides, dragomabin (**141**) and dragonamide B (**143**), along with the known metabolites carmabin A (**144**) and dragonamide A (**142**) (Figure 21) [71,72,73]. Carmabin A, dragomabin, and dragonamide A were tested against the W2 chloroquine-resistant malaria strain, initially showing IC_50_ values of 1.4, 21.0, and 10.7 µM for carmabin A, dragomabin, and dragonamide A, respectively. Subsequent side-by-side testing gave IC_50_ values of 4.3, 6.0, and 7.7 μM, indicating no significant difference in their antimalarial activity. However, carmabin A was more cytotoxic to Vero cells than the other compounds, making dragomabin the most selective between parasite and mammalian cells. The higher cytotoxicity of carmabin A is likely due to its longer aliphatic chain. Additionally, dragonamide B, tested later, showed no antimalarial activity, suggesting that an aromatic amino acid at the carboxy terminus is essential for activity in this compound series. In addition, the nonaromatic ring-containing alkynoic lipopeptide, jamaicamide B (**146**) (Figure 21), exhibited weak antimalarial activity (IC_50_ = 18.4 μM) with similar cytotoxicity (IC_50_ = 16.2 μM) to Vero cells. However, the terminal bromoacetylene homologue, jamaicamide A (**145**) (Figure 21), was inactive in this assay.

#### 5.1.9. Ikoamide

**Figure 22:**
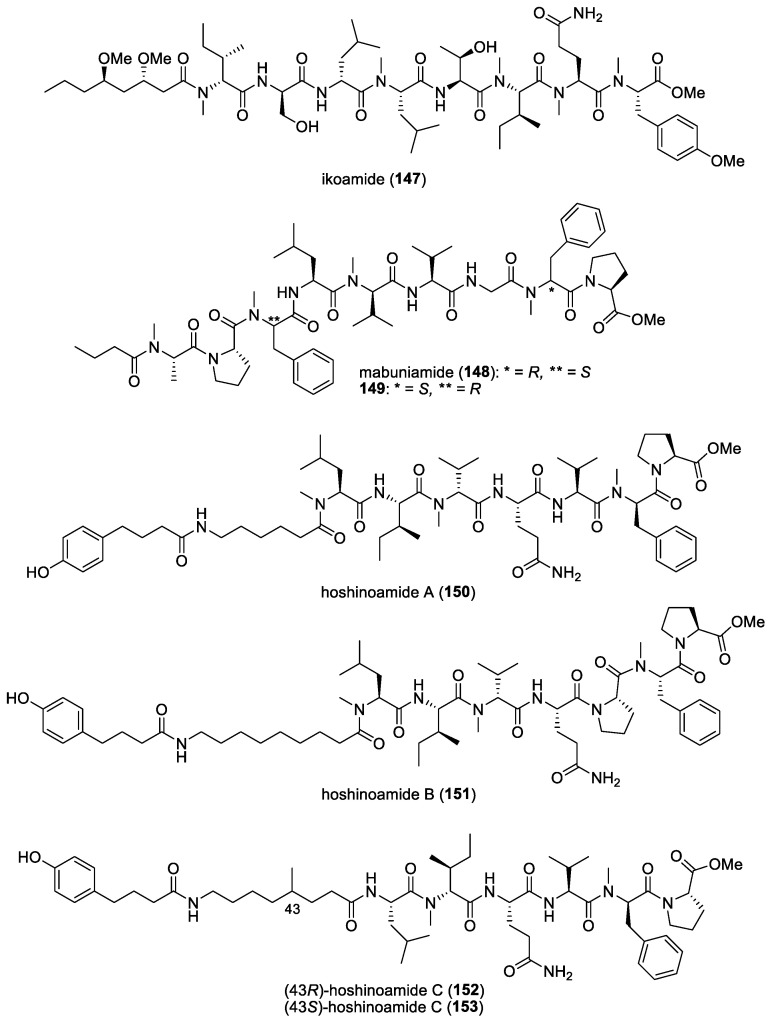
Antiparasitic marine cyanobacterial molecules.

An antimalarial lipopeptide, ikoamide (**147**) (Figure 22), was isolated from a marine cyanobacterium of the *Okeania* species, collected in Iko-pier, Kuroshima Island, Okinawa, Japan [74]. The antimalarial activity of ikoamide against the asexual erythrocytic stage of the *P. falciparum* 3D7 clone, a standard reference strain sensitive to most antimalarials, was evaluated. Ikoamide exhibited strong antiplasmodial activity with an IC_50_ value of 0.14 μM. Conversely, at 10 μM, ikoamide did not show growth-inhibitory activity against HeLa or HL60 cells. These results indicate that ikoamide selectively inhibits the growth of malarial parasites.

To produce sufficient ikoamide for biological evaluations, Suenaga and co-workers developed a total synthesis method [203]. Starting from eight amino acid derivatives and (*R*)-1,2-epoxypentane, they successfully synthesized ikoamide through the longest linear sequence of 18 steps, achieving an overall yield of 1.1%. This method not only provides an efficient route for synthesizing ikoamide but also facilitates the synthesis of structurally related compounds for structure–activity relationship (SAR) studies.

#### 5.1.10. Mabuniamide

The bioassay-guided fractionation of the marine cyanobacterium *Okeania* sp., collected in Odo, Okinawa, led to the isolation of the lipopeptide mabuniamide (**148**) (Figure 22) [75]. To confirm the absolute configuration of mabuniamide, its total synthesis was carried out and its stereoisomer (**149**) was obtained. Additionally, the antimalarial activities of both compounds against the asexual erythrocytic stage of the *P. falciparum* 3D7 clone were assessed. Mabuniamide and its stereoisomer, **149**, showed antiplasmodial activity, with IC_50_ values of 1.4 μM and 2.8 μM, respectively. In comparison, the positive control, chloroquine, had an IC_50_ value of 7.6 nM.

#### 5.1.11. Hoshinoamides

From two separate collections of the marine cyanobacterium *Caldora penicillata*, from Hoshino and Ikei Island, Okinawa, a series of acyclic lipopeptides, hoshinoamides A (**150**)–C (**152**) (Figure 22), having antimalarial activity, were isolated [76,77]. Hoshinoamides A and B did not inhibit the growth of HeLa cells at 10 μM. When the antimalarial activities of hoshinoamides A and B against the asexual erythrocytic stage of the *Plasmodium falciparum* 3D7 clone were evaluated, they exhibited antiplasmodial activity, with IC_50_ values of 0.52 and 1.0 μM, respectively. The subsequent evaluation of hoshinoamide C and its synthetic epimer (**153**) against the malarial parasite *P. falciparum* and *Trypanosoma brucei rhodesiense* revealed their moderate toxicities against these organisms, with IC_50_ values ranging from 0.87 to 4.4 μM [77]. Additionally, the configuration at C-43 of hoshinoamide C did not affect its antiparasitic activities, which were similar to those of hoshinoamides A and B.

#### 5.1.12. Pemuchiamides

**Figure 23:**
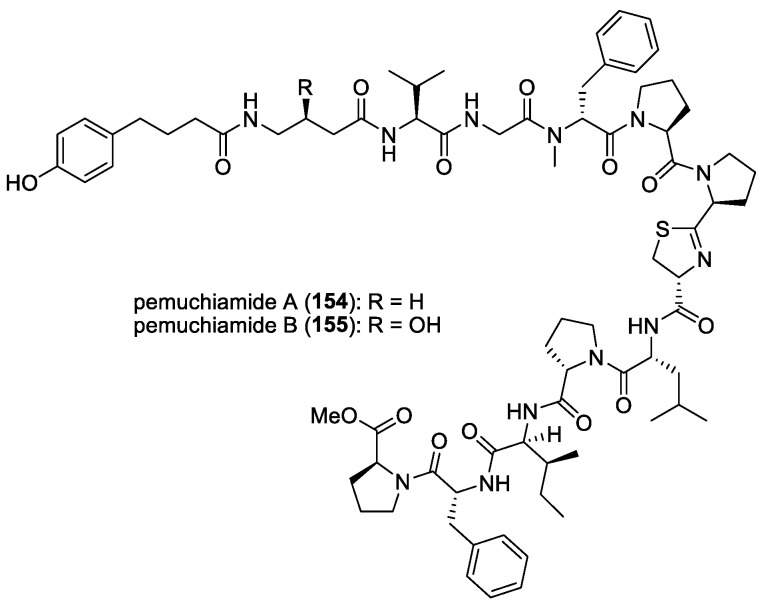
Antiparasitic marine cyanobacterial pemuchiamides.

Structurally related to hoshinoamides, new pemuchiamides A (**154**) and B (**155**) (Figure 23) were isolated from a marine cyanobacterium, *Hormoscilla* sp., collected on Pemuchi Beach, on Hateruma Island, Japan [78]. Despite the presence of a complex mixture of rotamers in chloroform-d, detailed analyses of their 2D NMR and tandem mass spectra successfully revealed their planar structures. Pemuchiamide A demonstrated strong growth-inhibitory activity against *Trypanosoma brucei rhodesiense*, with an IC_50_ of 0.63 μM, whereas pemuchiamide B exhibited activity which was 10 times weaker. This suggests that the hydroxy group at the C-3 position of the 4-aminobutanoic acid moiety adversely impacts antitrypanosomal activity. No antimalarial activity was reported for pemuchiamides.

#### 5.1.13. β-Hydroxy- and β-Amino-Containing Cyclic Depsipeptides

**Figure 24:**
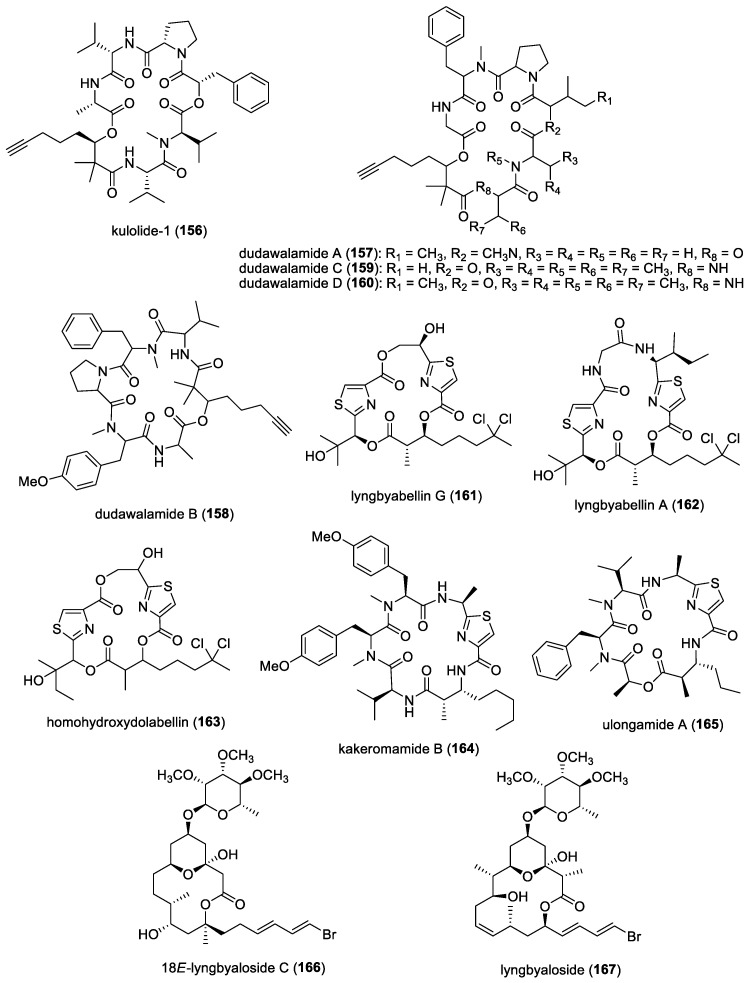
Marine cyanobacterial molecules with antiparasitic activity.

Several β-hydroxy- and β-amino-containing cyclic depsipeptides from marine cyanobacteria have been reported to possess anti-infective activities. A previously isolated cyclic depsipeptide, kulolide-1 (**156**) (Figure 24), was found to possess antimalarial activity against two malarial strains, the *P. falciparum* Dd2 clone and the 3D7 clone, with IC_50_ values of 1.62 and 1.49 μM, respectively [79]. Kulolide-1 was originally thought to be biosynthesized by the predatory cephalaspidean mollusk *Philinopsis speciosa* [204]. However, further investigations became necessary to establish the true biological origin of kulolide-1, upon the discovery that all other members in this superfamily were isolated exclusively from marine cyanobacteria. The predator–prey relationship established between the mollusk and marine cyanobacteria finally revealed the latter to be the true biosynthetic origin of the cyclic depsipeptide.

##### Dudawalamides

A family of cyclic depsipeptides containing 2,2-dimethyl-3-hydroxy-7-octynoic acid (Dhoya), named dudawalamides A (**157**)–D (**160**) (Figure 24), was isolated from a collection of the cyanobacterium *Moorena producens*, obtained near Dudawali Bay, Papua New Guinea [80]. The dudawalamides were tested for cytotoxic properties against the H-460 human lung cancer cell line and for antiparasitic properties against malaria, leishmaniasis, and Chagas disease. Dudawalamides A and D exhibited the strongest activity against *P. falciparum*, with IC_50_ values of 3.6 and 3.5 μM, respectively. However, their activities varied against the other parasites: dudawalamide A showed weaker activity against *Trypanosoma cruzi* and *Leishmania donovani*, while dudawalamide D was relatively potent against *L. donovani* (2.6 μM). However, dudawalamides B and C were significantly less potent against *P. falciparum*. These results indicate that minor changes in the configuration and sequence of residues significantly impact the bioactivity of these Dhoya-containing natural products.

##### Lyngbyabellins

The antimalarial activities of lyngbyabellins have been reported by two research groups. From two marine cyanobacterial strains of *Okeania* sp. and *M. bouillonii*, lyngbyabellins G (**161**) and A (**162**) (Figure 24) were active towards *P. falciparum* strain FCR-3 (IC_50_ 1.1 and 0.3 μM, respectively), while homohydroxydolabellin (**163**) (Figure 24) was moderately active (IC_50_ 6.4 μM) [81]. The antimalarial activity of lyngbyabellin A, along with other cyclic depsipeptides and macrolides, was also reported in another study by Sweeney-Jones et al., from a collection of *Moorena producens* from Fiji [82]. In their study, lyngbyabellin A was found to be more potent against *P. falciparum’s* blood stages, with an EC_50_ value of 0.15 nM, but less active against liver-stage *P. berghei*. Due to the potent cytotoxic nature of lyngbyabellins A and G, it was hypothesized that these molecules could act in the same way in erythrocytes, which skewed the results of the antimalarial assay. In the study by Sweeney-Jones et al., other compounds, such as kakeromamide B (**164**) (Figure 24) and ulongamide A (**165**) (Figure 24), displayed moderate activity against *P. falciparum’s* blood stages, with EC_50_ values of 0.89 and 0.99 μM, respectively, while kakeromamide B, 18*E*-lyngbyaloside C (**166**), and lyngbyaloside (**167**) (Figure 24) exhibited moderate liver-stage antimalarial activity against *P. berghei* liver schizonts, with EC_50_ values of 1.1, 0.71, and 0.45 μM, respectively. In addition, it was suggested that kakeromamide B may bind to several *Plasmodium* actin-like proteins and a sortilin protein, thereby causing possible interference with the parasitic invasion of host cells [82].

##### Veraguamides M and N

**Figure 25:**
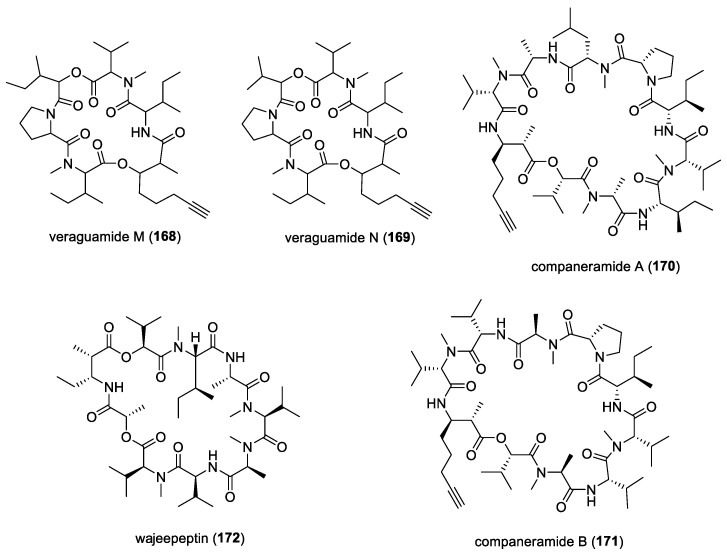
β-hydroxy/hydroxy-containing marine cyanobacterial molecules with antiparasitic activity.

A series of laboratory-based feeding preference assays for the sea hare *Dolabrifera nicaraguana* were conducted by offering six food options collected from nearby tidal pools in the Coiba National Park, Panama, which led to the isolation of new cyclic depsipeptides—veraguamides M (**168**) and N (**169**) (Figure 25)—from the preferred cyanobacterial food, i.e., cf. *Lyngbya* sp. [83]. In addition, veraguamides M and N showed in vitro activity against *P. falciparum,* with GI_50_ values of 4.2 and 4.3 μM, respectively, and therapeutic windows of 7.0–8.0 (based on moderate cytotoxicity to mammalian Vero cells, with GI_50_ values of 29.3 and 34.1 μM, respectively). Furthermore, veraguamide N was active against *Leishmania donovani*, with a GI_50_ value of 6.9 μM. However, it remains unknown whether these dietary-acquired secondary metabolites provide a chemical defense against protozoan parasites in mollusks.

##### Companeramides

From a coibamide A-containing marine cyanobacterial assemblage from Coiba Island, Panama, two new cyclic depsipeptides, companeramides A (**170**) and B (**171**) (Figure 25, were isolated [84]. Companeramides A and B exhibited no significant cytotoxicity at 1 μM against four human cancer cell lines, including NCI-H460 non-small-cell lung carcinoma, MDA-MB-231 breast adenocarcinoma, SF-295 glioblastoma, and SK-OV3 ovarian carcinoma cells. However, the initial antiparasitic activity observed in the parent fractions prompted the testing of the two pure compounds against three strains of the malaria parasite *P. falciparum,* using a fluorescence-based assay. The chloroquine-sensitive D6 strain was about twice as sensitive to companeramide A compared to the chloroquine-resistant Dd2 and 7G8 strains, with IC_50_ values of 0.57, 1.0, and 1.1 μM, respectively. In contrast, companeramide B exhibited similar activity against both the chloroquine-sensitive D6 strain (IC_50_ = 0.22 μM) and the chloroquine-resistant Dd2 strain (IC_50_ = 0.23 μM), but it was approximately three times less active against the chloroquine-resistant 7G8 strain. No compound demonstrated activity comparable to the chloroquine control against the chloroquine-sensitive D6 strain or the chloroquine-insensitive Dd2 and 7G8 strains.

##### Wajeepeptin

Recently, wajeepeptin (**172**) (Figure 25), a novel cyclic depsipeptide, was isolated from a marine cyanobacterium, *Moorena* sp., obtained from the Wajee Coast, Ie Island, Okinawa [85]. Its structure was determined through a combination of spectroscopic analyses, X-ray diffraction, and degradation reactions. Wajeepeptin exhibited moderate cytotoxicity against HeLa cells (IC_50_ = 3.7 μM) and demonstrated potent antitrypanosomal activity against *Trypanosoma brucei rhodesiense,* with an IC_50_ value of 0.73 μM. No antimalarial activity was reported for wajeepeptin.

#### 5.1.14. Venturamides A and B

**Figure 26:**
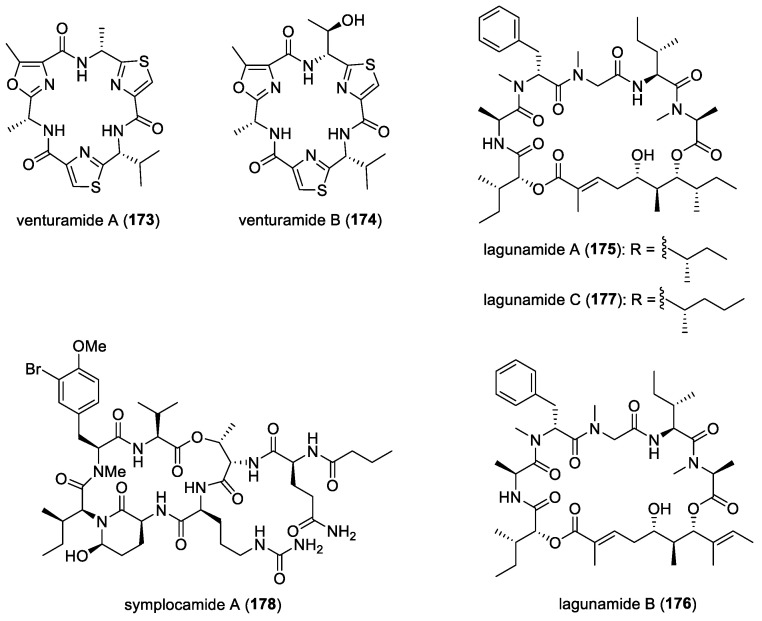
Marine cyanobacterial molecules with antiparasitic activity.

Two new heteroaromatic-containing cyclic hexapeptides, venturamides A (**173**) and B (**174**) (Figure 26), were isolated from the marine cyanobacterium *Oscillatoria* sp., collected from a shallow sandy inlet in Buenaventura Bay, Portobelo National Marine Park, Panama [86]. Venturamides A and B were tested for their antimalarial activity against the W2 chloroquine-resistant strain of the malaria parasite. Compounds **173** and **174** demonstrated strong in vitro activity against *Plasmodium falciparum* (8.2 μM and 5.6 μM, respectively), with only mild cytotoxicity to mammalian Vero cells (86 μM and 56 μM, respectively), resulting in a significant difference in activity between the parasite and host cells. Both compounds showed only mild activity when tested against *Trypanosoma cruzi* and *Leishmania donovani*.

#### 5.1.15. Lagunamides

Three cytotoxic cyclic depsipeptides, lagunamides A (**175**)–C (**177**) (Figure 26), were isolated from *Lyngbya majuscula*, collected from Pulau Hantu, Singapore [87,88]. These lagunamides have a planar macrocyclic scaffold composed of peptide and polyketide substructures, with the main variations occurring in the polyketide part. Lagunamides A and B are 26-membered macrocycles, whereas lagunamide C has an additional methylene carbon in its polyketide structure. The recent total synthesis of lagunamide C resulted in the structural revision of this molecule into the related analog odoamide [205]. Lagunamides A–C demonstrated potent activity against the *P. falciparum* NF54 strain, with IC_50_ values of 0.19, 0.91, and 0.29 μM, respectively. The double bond in the side chain of lagunamide B might account for its lower activity.

#### 5.1.16. Symplocamide A

The chemical investigation of *Symploca* sp. obtained from Sunday Island, Papua New Guinea, led to the purification of a cytotoxic Ahp-containing cyclic depsipeptide, symplocamide A (**178**) (Figure 26) [89]. In addition, symplocamide A was evaluated against three tropical parasites and showed significant antimalarial activity against W2 *P. falciparum,* with an IC_50_ of 0.95 µM, and moderate activity against *Trypanasoma cruzi* and *Leishmania donovani,* with IC_50_ values >9.5 µM. Its total synthesis was completed by Stolze and co-workers via solid-phase synthesis through the application of the masked glutamic aldehyde moiety [206].

### 5.2. Antitrypanosomal and Antileishmanial Molecules

#### 5.2.1. Kagimminols A and B

**Figure 27:**
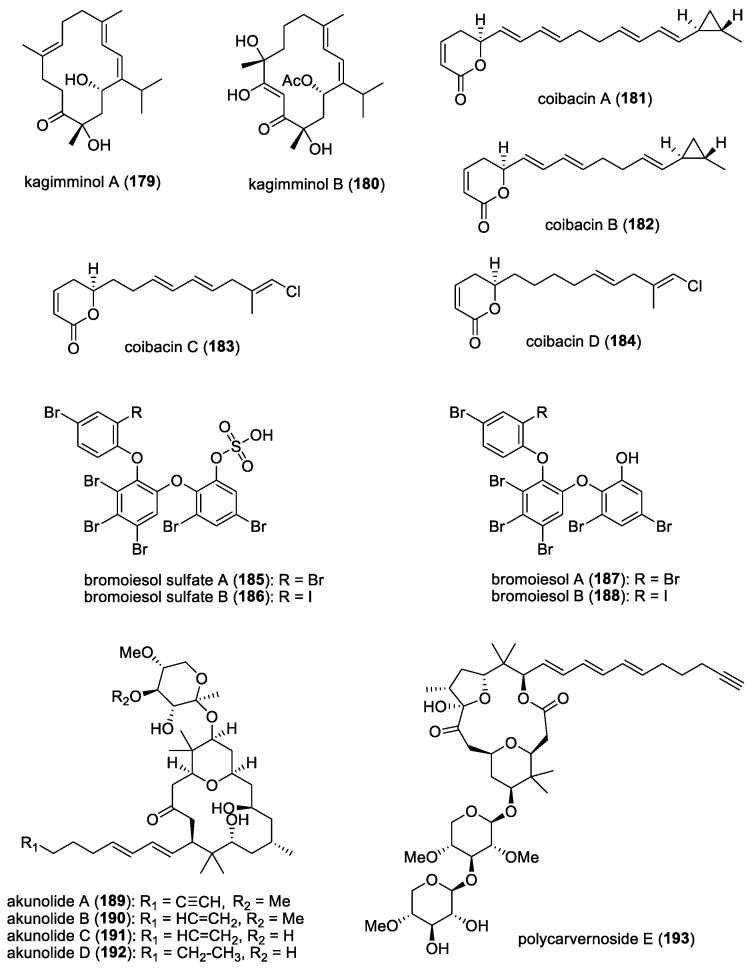
Antiparasitic marine cyanobacterial molecules.

New cembrene-type diterpenoids, kagimminols A (**179**) and B (**180**) (Figure 27), were isolated from an *Okeania* sp. marine cyanobacterium collected near Kagimmi Beach in Okinawa [90]. Utilizing DP4 analysis alongside an efficient NMR chemical shift calculation protocol, the relative configurations of kagimminols A and B were determined while their absolute configurations were established by comparing theoretical electronic circular dichroism (ECD) spectra with experimental data. In addition, kagimminols A and B exhibited moderate selective growth-inhibitory activity against the causative agent of human African trypanosomiasis, *Trypanosoma brucei rhodesiense* strain IL-1501, with IC_50_ values of 10 and 3.4 µM, respectively.

#### 5.2.2. Anaephene B

Based on generous gifts of anaephene B (**26**) (Figure 5) and two other synthetic analogs—**28** and **29** (Figure 5)—from Dr. Jonathan Mills, who previously discovered their antibiotic activities, the potency of these molecules was evaluated against *Leishmania tarentolae* by Zaman and co-workers [33]. The natural product anaephene B and the two synthetic analogs demonstrated effectiveness at levels comparable to meglumine antimoniate and amphotericin B, currently used to treat *Leishmania* infections. In addition, all three test compounds were as effective, if not more so, in inhibiting the viability of *Leishmania tarentolae* compared to MRSA, with minimum inhibitory concentration values of 3.64 (**26**), 1.22 (**28**), and 3.68 (**29**) μg/mL. Moreover, the test compounds had minimal impact on detectable secreted acid phosphatase activity, suggesting that they may not alter the infectivity potential of parasites not inhibited by this class of potential drugs.

#### 5.2.3. Coibacins

Four unsaturated polyketide lactone derivatives, named coibacins A (**181**)–D (**184**) (Figure 27), were isolated from a Panamanian marine cyanobacterium, cf. *Oscillatoria* sp., collected from rocks in a bay near Uvas Island, Coiba National Park [91]. These compounds were tested for activity against tropical diseases, which revealed that coibacin A exhibited potent activity against *Leishmania donovani* axenic amastigotes, with an IC_50_ value of 2.4 μM, while the other coibacins were slightly less active. This antileishmanial activity was also confirmed with *L. mexicana* axenic amastigotes. However, in a macrophage assay with *L. mexicana*, the coibacins were inactive, likely due to an inability to cross the cell membrane of these cells. Out of the various coibacins, coibacin A was the least cytotoxic against NCI-H460 human lung cancer cells (IC_50_ = 31.5 μM). The absolute stereochemistry of coibacin A was subsequently confirmed by total synthesis, which involved 12 steps and resulted in a 3.4% overall yield [207]. Natural coibacin A was determined to have a 5*R*,16*S*,18*S* absolute configuration based on the synthesis of its stereoisomers. The correct isomer of coibacin B was also synthesized using the configuration assignment of coibacin A.

#### 5.2.4. Bromoiesol Sulfates

Polyhalogenated aryl ethers, bromoiesol sulfates A (**185**) and B (**186**) (Figure 27), along with their hydrolysates (**187** and **188**), were isolated from the marine cyanobacterium *Salileptolyngbya* sp., collected on Ie-Island, Okinawa [92]. Their structures were determined through a small-molecule accurate recognition technology (SMART) analysis of HMQC data for bromoiesol A and single-crystal X-ray diffraction analyses for bromoiesols A and B. The structures were further verified by synthesizing bromoiesol sulfate A and bromoiesol A. In general, the biosynthetic pathways for polyhalogenated aryl ethers, as elucidated by Moore’s group, show that each aromatic ring originates from 4-hydroxybenzoic acid, brominated by the enzyme Bmp5, with the 4-hydroxybenzoic acid being synthesized from chorismic acid by chorismate lyase Bmp6. The aryl ether bonds are then formed by the cytochrome P450 enzyme Bmp7 [208].

The bromoiesol compounds demonstrated antitrypanosomal activity against the *Trypanosoma brucei rhodesience* IL-1501 strain, without affecting HeLa cell growth at a concentration of 10 μM [92]. While iodine substitution did not alter their activity, hydrolysis of the sulfate group significantly increased their antiparasitic activity. Due to the instability of the sulfate group and the notable difference in activity between the sulfates (bromoiesol sulfates A and B) and their hydrolysates (bromoiesols A and B), it is suggested that bromoiesol sulfates could be prodrugs in the natural environment.

#### 5.2.5. Akunolides and Polycavernoside E

From a collection of *Okeania* sp. obtained from Akuna Beach, Okinawa, a series of 16-membered macrolide glycosides, akunolides A (**189**) to D (**192**) (Figure 27), were reported [93]. The akunolides showed moderate antitrypanosomal activity against *Trypanosoma brucei rhodesiense*, with IC_50_ values ranging from 11 to 14 μM. Interestingly, akunolides A and C showed no cytotoxic activity against normal human WI-38 cells, suggesting that the presence of a terminal alkyne unit and xylose demethylation at C-3′ could be essential in an akunolide analog for better selective toxicity against the parasite. A related molecule, polycavernoside E (**193**) (Figure 27), was subsequently isolated from the same marine cyanobacterium, *Okeania* sp., obtained from Akuna Beach in 2022 [94]. Polycavernoside E showed moderate antitrypanosomal activity against *Trypanosoma brucei rhodesiense,* with an IC_50_ value of 9.9 μM.

#### 5.2.6. Hennaminal and Hennamide

**Figure 28:**
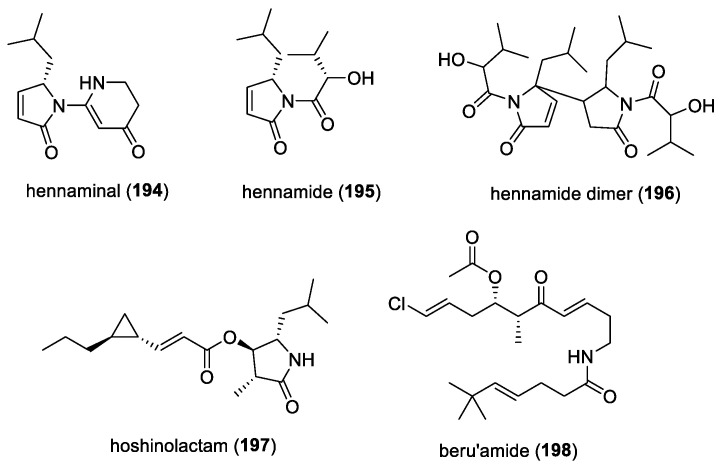
Marine cyanobacterial molecules with antiparasitic activity.

Two new secondary metabolites, hennaminal (**194**) and hennamide (**195**) (Figure 28), were purified from the marine cyanobacterium *Rivularia* sp., collected from a coral reef in Higashihennazaki, Miyako Island, Okinawa [95]. Hennaminal contains a rare β,β-diamino unsaturated ketone functional group, while hennamide possesses a reactive *N*-acyl pyrrolinone moiety, which promotes self-dimerization to form a hennamide dimer (**196**) (Figure 28). Due to the instability of hennamide, its absolute stereochemistry was determined by total synthesis. Hennaminal and synthetic hennamide were evaluated for their cytotoxicity and antiparasitic activity. Both compounds exhibited minimal inhibition of HeLa cell growth, with IC_50_ values of 140 μM and 22 μM, respectively. In addition, they showed moderate growth-inhibitory activity against the bloodstream form of *Trypanosoma brucei rhodesiense,* with IC_50_ values of 11 μM and 9.7 μM for hennaminal and hennamide, respectively.

#### 5.2.7. Hoshinolactam

In the quest for new antiprotozoal compounds, hoshinolactam (**197**) (Figure 28), an antitrypanosomal lactam, was isolated from a marine cyanobacterium off the coast near Hoshino, Okinawa [96]. Its gross structure was elucidated through spectroscopic analyses, and its absolute configuration was confirmed by the first total synthesis. Hoshinolactam exhibited potent antitrypanosomal activity against the *Trypanosoma brucei brucei* GUTat 3.1 strain, with an IC_50_ value of 3.9 nM, showing no cytotoxicity against human fetal lung fibroblast MRC-5 cells. To discover novel antitrypanosomal agents based on hoshinolactam, Reddy and colleagues synthesized and evaluated 14 different analogs of the natural product using various combinations of acids and lactams [209]. However, antitrypanosomal activity assays revealed that the synthesized analogs were less potent than the parent natural product.

#### 5.2.8. Beru’amide

A 68 μg quantity of an acyclic polyketide, named beru’amide (**198**) (Figure 28), was isolated from a marine cyanobacterium, *Okeania* sp., collected at Beru, Kasari-cho, Kagoshima, Japan [97]. Using several advanced techniques, including DFT-based chemical shift calculations, the structure of the molecule was successfully determined. Moreover, the total synthesis of this highly functionalized natural product was accomplished. Beru’amide exhibited moderate growth-inhibitory activity against HeLa cells (IC_50_ = 8.0 μM) and potent antitrypanosomal activity against *Trypanosoma brucei rhodesiense* (IC_50_ = 1.2 μM).

#### 5.2.9. Gallinamide A (= Symplostatin 4)

**Figure 29:**
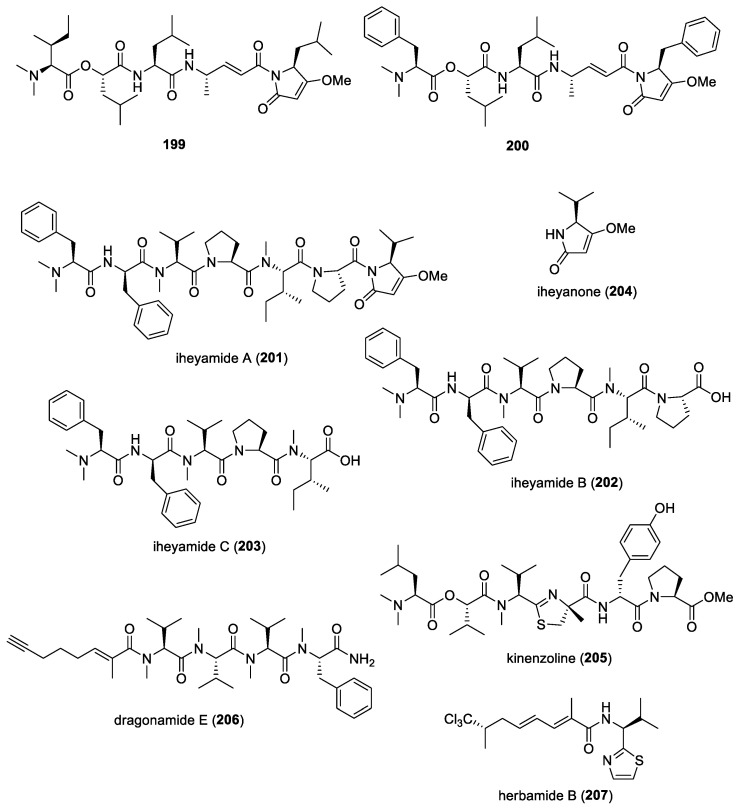
Antiparasitic marine cyanobacterial molecules.

Gallinamide A (**10**) (Figure 2), initially identified for its modest antimalarial activity, was later found to be a potent, selective, and irreversible inhibitor of the human cysteine protease cathepsin L [14]. Cathepsin L is a member of the cathepsin enzyme family with endopeptidase activity and plays critical roles in various cellular functions. Based on substrate selectivity, human cathepsins L and V align closely with cruzain (from *Trypanosoma cruzi*) and cathepsin L (from *Leishmania mexicana*), suggesting that inhibitors of human cathepsin L could be effective against these parasitic enzymes. Using gallinamide A as a structural template, Gerwick and co-workers produced the most potent gallinamide analog against human cathepsin L (*K*_i_ = 0.0937 nM, *k*_inact_/*K*_i_ = 8,730,000) [17]. Given its structural similarity and substrate preference to cruzain, gallinamide A and its analogs were found to be highly effective inhibitors of cruzain and toxic to *T. cruzi* in the intracellular amastigote stage. The most effective compound, **199** (Figure 29), with an IC_50_ of 5.1 nM, showed low activity against insect-stage epimastigote and host cells, marking it a promising candidate for Chagas disease treatment [17].

In a subsequent study, Gerwick and co-workers assessed the effectiveness of gallinamide A and over 20 synthetic analogs against intracellular *Trypanosoma cruzi* amastigotes and the cysteine protease cruzain [210]. In this process, they successfully determined the co-crystal structures of cruzain with gallinamide A and two analogs at ~2 Å resolution. Structure–activity relationship (SAR) data revealed that the *N*-terminal end of gallinamide A is loosely bound and contributes minimally to drug–target interactions. At the C-terminus, intramolecular π-π stacking interactions between the aromatic substituents at P1′ and P1 help maintain the bioactive conformation of the inhibitors, reducing entropic loss during target binding. In addition, molecular dynamics simulations revealed that, without an aromatic group at P1, the P1′ substituent interacts with tryptophan-184. While the P1-P1′ interactions did not affect the anti-cruzain activity, they enhanced the anti-*T. cruzi* potency by approximately 5-fold, likely caused by the improved solubility and permeability of the analogs. Their approach of integrating structural data with the per-residue free energy decomposition information facilitates the computational validation of new chemical modifications before synthesizing future gallinamide A analogs.

In another recent development, Mares and co-workers screened a library of 19 synthetic gallinamide A analogs and identified nanomolar inhibitors of the cathepsin B-type protease SmCB1, which is a drug target for the treatment of *Schistosoma mansoni* [211]. Specifically, the gallinamides induced a variety of harmful phenotypic effects against cultured *S. mansoni* schistosomula and adult worms, including slowed motility, uncoordinated movements, and damaged teguments. In addition, imaging with a fluorescent activity-based probe derived from gallinamide A showed that SmCB1 is the primary target of gallinamides in the parasite, which was confirmed via high-resolution crystal structures of SmCB1 in a complex with gallinamide A and its two analogs, **199** and **200**, revealing the acrylamide covalent warhead and its binding mode within the active site. Furthermore, quantum chemical calculations assessed the contribution of individual positions in the peptidomimetic scaffold to target inhibition, highlighting the significance of the P1′ and P2 positions within gallinamides. Results from this study highlight future directions for the better design of gallinamides as potential drug agents for the treatment of schistosomasis.

#### 5.2.10. Iheyamides

Iheyamides A (**201**)–C (**203**) (Figure 29), newly identified linear peptides, were isolated from a marine *Dapis* sp. cyanobacterium collected on Noho Island, Okinawa [98]. Iheyamide A exhibited moderate antitrypanosomal activity against *Trypanosoma brucei rhodesiense* and *Trypanosoma brucei brucei,* with an IC_50_ value of 1.5 μM, while the other two analogs, iheyamides B and C, did not. The cytotoxicity of iheyamide A against normal human WI-38 cells was ten times weaker than its antitrypanosomal activity. The structure–activity relationship analysis revealed that an isopropyl-*O*-Me-pyrrolinone moiety was essential for antitrypanosomal activity. Additionally, Suenaga and co-workers isolated this pyrrolinone moiety as a new natural product from a marine cyanobacterium, naming it iheyanone (**204**) (Figure 29) [99]. As anticipated, iheyanone exhibited antitrypanosomal activity, albeit with less potency than iheyamide A. To further elucidate the structure–activity relationships, Suenaga and co-workers completed the total synthesis of iheyamide A and iheyanone and assessed the antitrypanosomal activities of various synthetic intermediates. They found that the longer the peptide chain, the more pronounced the antitrypanosomal activity.

#### 5.2.11. Kinenzoline

Kinenzoline (**205**) (Figure 29), a newly discovered linear depsipeptide, was isolated in small amounts from a marine *Salileptolyngbya* sp. cyanobacterium collected on Kinenhama Beach, Kagoshima, Japan [100]. Subsequently, the total synthesis of the natural product was carried out to confirm its structure and produce sufficient quantities for biological evaluation. The antitrypanosomal activity of kinenzoline against the *T. b. rhodesiense* strain IL-150115 and its cytotoxicity against WI-38 cells (normal human fibroblasts) were evaluated. Natural kinenzoline demonstrated moderate growth-inhibitory activity against *T. b. rhodesiense* (IC_50_ = 5.0 μM) without cytotoxicity against WI-38 cells at 20 μM, indicating that kinenzoline possesses highly selective toxicity against the parasite.

#### 5.2.12. Dragonamides A and E and Herbamide B

Bioassay-guided isolation from an antileishmanial extract of *Lyngbya majuscula*, collected from mangrove roots in the Bastimentos National Park, Bocas del Toro, Panama, resulted in the isolation of dragonamide E (**206**) (Figure 29), along with two known modified linear peptides, dragonamide A (**142**) and herbamide B (**207**) [101]. Dragonamides A and E and herbamide B demonstrated antileishmanial activity, with IC_50_ values of 6.5, 5.1, and 5.9 μM, respectively. In contrast, dragonamide B showed no activity against the tested parasites, suggesting that the presence of aromatic ring-containing residue at the peptide terminus is crucial for this kind of activity [101].

#### 5.2.13. Almiramides

**Figure 30:**
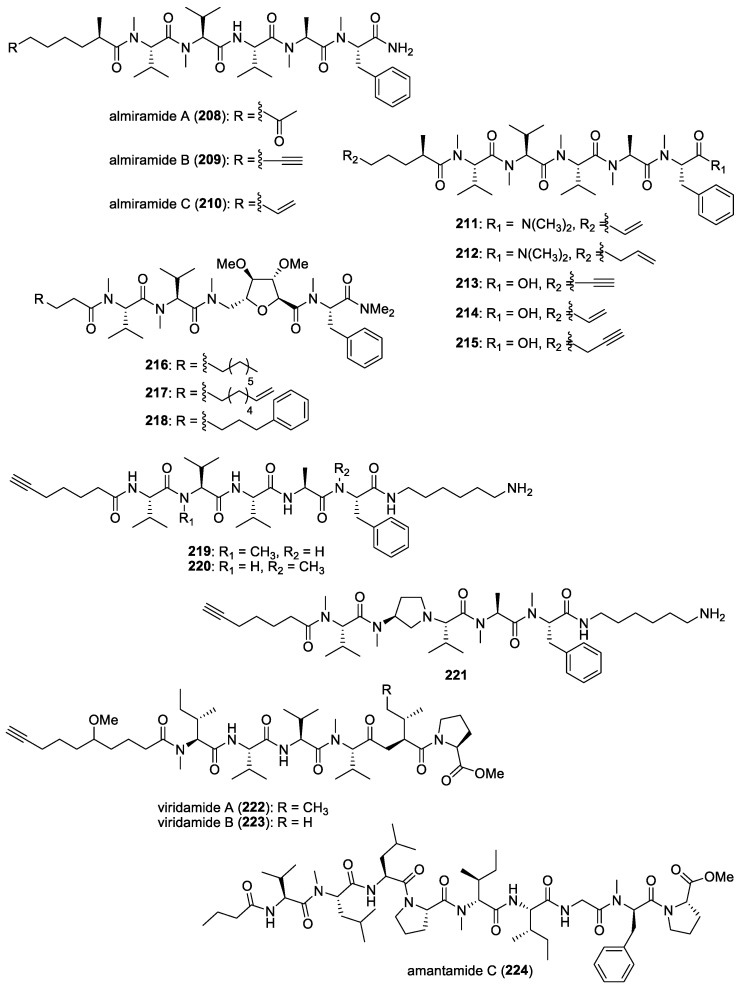
Antiparasitic marine cyanobacterial molecules.

Almiramides A (**208**)–C (**210**) (Figure 30) are *N*-methylated linear lipopeptides isolated from the marine cyanobacterium *Lyngbya majuscula*, collected from mangrove roots on a small island in the Bocas del Toro National Marine Park, Panama [102]. The biological evaluation of these linear lipopeptides revealed that almiramides B and C exhibited strong in vitro antiparasitic activity against *L. donovani*, with IC_50_ values of 2.4 and 1.9 μM, respectively. In contrast, almiramide A was completely inactive at concentrations up to 13.5 μM, suggesting that an unsaturated terminus on the side chain is essential for activity. These almiramides were also evaluated for their activity against both *Plasmodium falciparum* (W2 chloroquine-resistant strain) and *Trypanosoma cruzi*. However, they were found to be inactive at the highest tested concentrations (13.5 μM) in all instances. The subsequent semi-synthesis of almiramide analogs, using the solid-phase peptide synthesis (SPPS) methodology, led to the discovery of synthetic compounds (e.g., **211**) (Figure 30) with superior in vitro activity and improved selectivity profiles compared to the natural products (e.g., **212**–**215**) (Figure 30). These almiramide derivatives are promising candidates for developing leishmaniasis treatments, partly due to their probable mechanism of action involving the disruption of vital energy machinery proteins in the glycosome [103]. The glycosome is a peroxisome-related organelle essential for metabolic processes in trypanosomatids and lacks a mammalian counterpart [212]. In fact, an analysis of almiramide analogs through affinity capture and fluorescent microscopy experiments on *T. brucei* revealed that the glycosome protein PEX11 and the glycosomal integral membrane protein-5a (GIM5A) are probable targets.

The active conformation of almiramide has been further explored by substituting the Val^3^-Ala^4^ dipeptide segment with a mannose-derived sugar amino acid (MAA) in analogs **216**–**218** (Figure 30). These sugar–peptide hybrids demonstrated comparable activity and selectivity indexes to miltefosine, a second-line antileishmanial drug, against intra-macrophage amastigotes of *L. donovani* and Vero cells [213]. In addition, SAR and NMR studies indicated that, among all the synthesized compounds, the MAA-containing permethylated analogs with longer hydrophobic chains at the *N*-terminus were more active than their unmethylated counterparts, likely due to the improved cell permeability of the former analogs.

In another study by Lubell and co-workers, a structure–activity relationship study was carried out to examine the influence of *N*-methylation and turn-inducing a-amino g lactam (Agl) and *N*-aminoimidazalone (Nai) residues within almiramide peptides against various strains of *L. infantum* (including WT, Sb2000.1, AmB1000.1, and MF200.5) and on cytotoxicity [104]. The synthesis and biological evaluation of twenty-five analogs revealed that derivatives with a single methyl group on either the first (e.g., **219**) or fifth residue amide nitrogen (e.g., **220**) exhibited greater activity than the permethylated peptides and showed a relatively high potency against resistant strains of *L. infatum*. Moreover, replacing the amino amide residues in the peptide with turn-inducing Agl and Nai counterparts generally reduced their antiparasitic activity. However, peptide amides with Agl residues at the second position (e.g., **221**) retained significant potency in both the unmethylated and permethylated series. Their study showed that conformers extended around the central residues and mobility at the peptide’s extremities may enhance almiramide activity.

#### 5.2.14. Viridamides

A chemical and phylogenetic investigation of a cultured marine cyanobacterium, *Oscillatoria nigro-viridis*, from Panama resulted in the isolation of two new PKS-NRPS-derived molecules, viridamides A (**222**) and B (**223**) (Figure 30) [105]. Viridamide A was tested against a series of relevant tropical pathogens and cancer cell lines. Notably, **222** exhibited significant activity against the three parasitic protozoa *Trypanosoma cruzi* (IC_50_ = 1.1 μM), *Leishmania mexicana* (IC_50_ = 1.5 μM), and *Plasmodium falciparum* (IC_50_ = 5.8 μM), with minimal toxicity to the treated cancer cell lines.

#### 5.2.15. Amantamide C

Amantamides are lipopeptides that function as selective agonists for the CXC chemokine receptor 7 while influencing spontaneous calcium oscillations in primary cultured neocortical neurons [214,215]. Recently, Iwasaki and co-workers isolated a new analog, amantamide C (**224**) (Figure 30), from the marine cyanobacterium *Okeania* sp., collected on Tonaki Island, Japan [106]. The growth-inhibitory activity of amantamide C was assessed against HeLa and HL60 cells using an MTT assay, but it did not exhibit inhibitory effects on either cell line at 10 μM. However, HeLa cells treated with 50 μM of the molecule displayed spindle-like morphological changes after 24 h, and apoptosis-like cell death was observed after 48 h. In addition, amantamide C inhibited the growth of *Trypanosoma brucei rhodesiense,* with an IC_50_ value of 2.8 μM.

#### 5.2.16. Okeaniazole A

**Figure 31:**
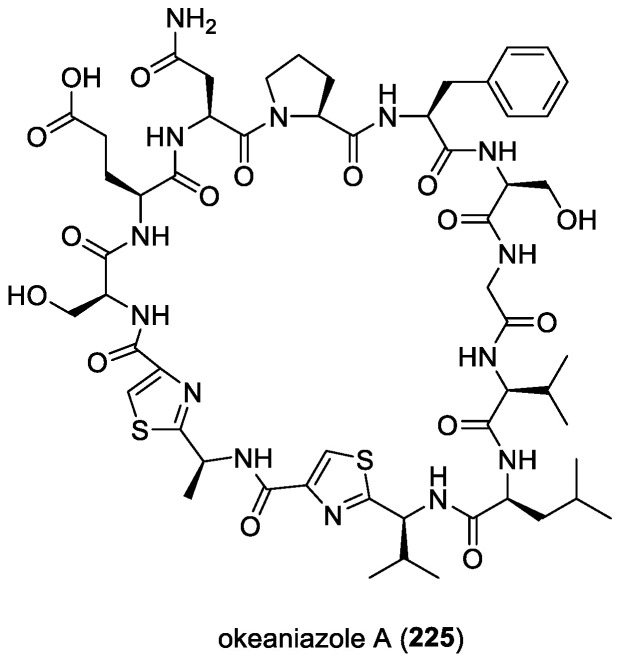
Antiparasitic okeaniazole A.

Recently, researchers have reported the isolation and structural elucidation of a novel thiazole-containing cyclic peptide, okeaniazole A (**225**) (Figure 31), from a bloom of the marine cyanobacterium *Okeania hirsute*, collected on Kuba Beach, Nakagusuku, Okinawa [19]. Based on its structure, okeaniazole A is classified within the cyanobactin family. Okeaniazole A and dolastatin 3 (**11**) (Figure 2), previously isolated from the same cyanobacterial strain, were also tested for antileishmanial activity due to their structural similarity. Okeaniazole A and dolastatin 3 exhibited similar inhibitory activity against the parasitic protozoan *Leishmania major*, with IC_50_ values of 12.1 and 12.5 μM, respectively. The authors noted that the presence of two thiazole rings in these cyclic structures could be a factor influencing their biological activity.

#### 5.2.17. Janadolide

**Figure 32:**
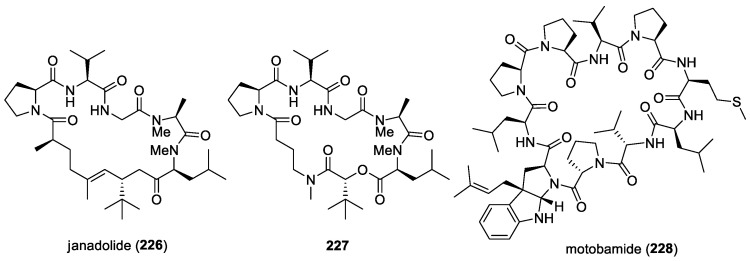
Antiparasitic marine cyanobacterial molecules.

Janadolide (**226**) (Figure 32), a new cyclic polyketide–peptide hybrid with a *tert*-butyl group, was isolated from an *Okeania* sp. marine cyanobacterium collected near Janado, Okinawa [107]. In biological activity tests, janadolide demonstrated potent antitrypanosomal activity against the *Trypanosoma brucei brucei* GUTat 3.1 strain (the causative agent of Nagana disease in animals), with an IC_50_ value of 47 nM, which is more potent than the commonly used therapeutic drug suramin. Additionally, janadolide did not inhibit the growth of human cells, such as MRC-5, HL60, and HeLa cells, even at 10 μM. Its first total synthesis was achieved by Suenaga and co-workers [216]. Subsequent synthetic efforts by Reddy and co-workers showed that the *tert*-butyl group in janadolide is essential for its antitrypanosomal activity [217]. In addition, several simplified analogs (e.g., **227**) of janadolide were synthesized using solid-phase synthesis and exhibited moderate micromolar-range antitrypanosomal activity against *Trypanosoma brucei rhodesiense* and *T. cruzi* parasites [108]. However, both the natural product and synthetic analogs were not active against *L. donovani*. Structure–activity data from the study showed a relatively narrow range of IC_50_ values (33–104 μM), indicating that replacing the olefin group and ester linkage with amide bonds did not significantly affect activity. It was hypothesized that the methyl groups at selected positions may play a key role in stabilizing the natural product’s 3D conformation. More importantly, none of the synthetic molecules showed any cytotoxicity against human L6 cell lines (up to a concentration of 100–150 μM), unlike the clinically approved drug melarsoprol.

#### 5.2.18. Motobamide

Motobamide (**228**) (Figure 32), a new cyclic peptide containing a *C*-prenylated cyclotryptophan residue, was isolated from a marine *Leptolyngbya* sp. cyanobacterium obtained from Bise, Okinawa [109]. Motobamide inhibited the growth of the bloodstream form of *T. b. rhodesiense*, the causative organism of human African sleeping sickness, with an IC_50_ value of 2.3 μM. Its cytotoxicity against WI-38 cells, normal human fibroblasts, was significantly lower, showing a more than 20-fold-weaker activity.

### 5.3. Molluscicidal Compounds

**Figure 33:**
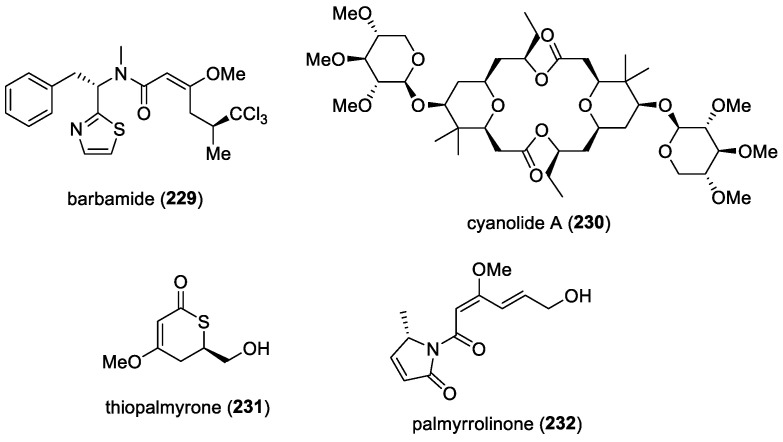
Molluscicidal marine cyanobacterial molecules.

Schistosomiasis is one of thirteen neglected tropical diseases characterized by high morbidity and mortality, collectively impacting one billion of the world’s poorest people, primarily in developing countries [218]. Molluscicides are vital for controlling schistosomiasis, as snails of the genus *Biomphalaria* act as intermediate hosts for the trematode parasite *Schistosoma*. Several marine cyanobacterial compounds have been reported to exhibit significant molluscicidal properties against *B. glabrata*. For instance, one of the first marine cyanobacterial compounds reported to exhibit molluscicidal activity (LC_100_ = 100 μg/mL) against *B. glabrata* was barbamide (**229**) (Figure 33), isolated from a collection of *L. majuscula* from Barbara Beach, Curacao, in 1996 [110]. In addition to antifungal activity, tanikolide (**17**) demonstrated molluscicidal activity against *B. glabrata,* with a median lethal dosage (LD_50_) of 9.0 μg/mL [25]. Cyanolide A (**230**) (Figure 33), a glycosidic macrolide, isolated from *Lyngbya bouillonii* obtained from Papua New Guinea, possesses a highly potent molluscicidal agent against *B. glabrata*, with an LC_50_ value of 1.2 μM [111]. Subsequent biological evaluations revealed that the glycosidic macrolide was relatively noncytotoxic against the H-460 human lung adenocarcinoma and Neuro-2a mouse neuroblastoma cell lines. Due to the unique structure of cyanolide A, at least nine papers have been reported on its total synthesis [219,220,221,222,223,224,225,226,227]. Lastly, thiopalmyrone (**231**) and palmyrrolinone (**232**) (Figure 33), isolated from extracts of a Palmyra Atoll assemblage of two cyanobacteria, cf. *Oscillatoria* and *Hormoscilla* sp., represent new and potent molluscicidal chemotypes against *B. glabrata*, with LC_50_ values of 8.3 and 6.0 μM, respectively [112]. When tested as an equimolar mixture, only a slight enhancement in the molluscicidal effect (LC_50_ = 5.0 μM) was observed.

## 6. Conclusions

This comprehensive review on anti-infectives reported from marine cyanobacteria presented more than 200 specialized molecules. A majority of these molecules belong to either peptides or a hybrid polyketide–peptide structural class. Of the various anti-infective compounds, the therapeutic area where marine cyanobacterial compounds have made the most impact is perhaps antiparasitic therapy. Highly potent molecules having nanomolar/picomolar activities, such as bastimolides, palstimolide A, janadolide, almiramide, gallinamide A, anaephene B, and carmaphycin B, have been uncovered as drug leads. Additionally, several potent synthetic analogs based on natural product templates have been synthesized as potential antiparasitic drug candidates. Several key features, such as their relatively weak cytotoxicity to eukaryotic cells, selectivity, and intracellular targets within parasites, have made them attractive sources of antiparasitic drug agents. However, only a limited number of marine cyanobacterial molecules have been tested for in vivo activity against microbial parasites. As a result, the chemical synthesis of the most promising identified compounds and their analogs is essential for evaluating their effects on various stages of parasites using in vivo animal models.

From an ecological perspective, certain marine invertebrates, such as nudibranchs, are known to sequester cyanobacterial molecules by feeding on these microalgae in nature. It would be interesting to explore whether these sequestered molecules confer protection to marine invertebrates from microbial/parasitic infection. In addition, interkingdom signaling by structurally related marine cyanobacterial molecules has also been suggested [228]. For instance, highly oxygenated cyanobacterial compounds containing a five- or six-membered ring unit and an acyl chain of varying length, such as certain malyngamides, honaucins, coibacins, and tumonoic acids, are found to inhibit bacterial quorum sensing and have anti-inflammatory activity. It has been hypothesized that such cyanobacterial compounds with dual functions constitute an evolutionary advantage as they can interact with both prokaryotic and eukaryotic life forms. These molecules could reduce/prevent biofilm formation by microbial competitors and down-regulate the innate immune system of the marine invertebrates they may associate with. Furthermore, several cyanobacterial-derived modified free acids, such as lyngbic acid, lyngbyoic acid, and pitinoic acids, are able to modulate bacterial quorum sensing systems. Moreover, these modified fatty acids are found in high abundance and are incorporated by microalgae into larger molecules, such as malyngamides, via amide linkage. The presence of these modified fatty acids could inspire their use in forming various synthetic bioactive molecules via chemical synthesis. In summary, the development of novel anti-infective agents from marine cyanobacteria is looking bright, and, without doubt, concerted efforts by researchers will lead to clinical anti-infective drugs in the near future.

## Figures and Tables

**Table 1:** Anti-infective and molluscicidal agents derived from marine cyanobacteria.

Compound	Species/Location	Anti-Infective Activity	Ref.
Aplysiatoxin (**1**), debromoaplysiatoxin (**2**), and 3-methoxydebromoaplysiatoxin (**5**)	*Trichodesmium erythraeum*/ Seringat Island, Singapore	**2** and **5**: potent against CHIKV **1**: induces proviral expression up to 900-fold-lower concentrations compared to prostatin Synthetic **6** and **7**: work synergistically with JQ1 to reactivate proviral expression	[8,9,10,11]
Malyngamides (**8** and **9**)	Australian cyanobacterium	Weak anti-HIV activity	[12]
Gallinamide A (**10**)	*Schizothrix* sp./Caribbean coast of Panama	**10**: inhibition of SARS-CoV-2 with IC_50_ values in the low nanomolar to picomolar range; potent antimalarial activities; and moderate in vitro activity against *P. falciparum* and *Leishmania donovani* Synthetic **199**: inhibitor of cruzain and toxic to *T. cruzi* in the intracellular amastigote stage	[13,14,15,16,17]
Dolastatin 3 (**11**)	*Lyngbya majuscula*/Big Goby marine lake, Palau	Anti-HIV activities based on HIV-1 integrase inhibition assays and inhibitory activity against the parasitic protozoan *Leishmania major*	[18,19]
Cyanopeptolin CP978 (**12**)	*Nostoc edaphicum* CCNP1411 (culture)/Baltic Sea	Effective against three SARS-CoV-2 variants (Alpha, Micron, and Delta)	[20]
Divamides (**13** and **14**)	*Prochloron didemni*/Eastern Papua New Guinea	Anti-HIV activities	[21]
2-Hydroxyethyl-11-hydroxyhexadec-9-enoate (**15**)	*Leptolyngbya* sp. LT19/Gulf of Thailand	Effective against Gram-negative shrimp pathogens *Vibrio harveyi* and *V. parahaemolyticus*	[22]
MGDG-palmitoyl (**16**)	*Oscillatoria acuminata* NTAPC05/Mandapam, Ramanathapuram District, Tamil Nadu, India	Effective against ESBL bacterial producers	[23]
Tanikolide (**17**)	*Lyngbya majuscula*/Tanikeli Island, Madagascar	**17**: antifungal against *Candida albicans* and molluscicidal against *Biomphalaria glabrata* Synthetic **20**: activity against MRSA	[24,25]
Malyngolide (**22**)	*L. majuscula*/Kahala Beach, Oahu	**22**: active against *Mycobacterium smegmatis* and *Streptococcus pyogenes*; less active against *Staphylococcus aureus* and *Bacillus subtilis*; interferes with QS circuitry; inhibits elastase production by *Pseudomonas aeruginosa* PAO1; and inhibits growth of *Dendryphiella salina* and *Lindra thalassiae* Synthetic **23** and **24**: active against MRSA	[24,26,27,28,29]
Anaephenes A (**25**) and B (**26**)	*Hormoscilla* sp./Anae Island, Guam	**25** and **26**: moderately active against *B. cereus* and *S. aureus*; active against MRSA **26**: inhibits the viability of *Leishmania tarentolae* Synthetic **28** and **29**: active against MRSA and inhibit the viability of *Leishmania tarentolae*	[30,31,32,33]
Polybrominated diphenyl ethers (PBDEs)	Cyanobacterial symbiont *Hormoscilla spongeliae*/various locations	Diverse activities, including antibacterial and antifungal effects	[34,35]
Crossbyanol B (**33**)	*Leptolyngbya crossbyana*/Hawaiian coral reefs	Antibacterial against MRSA	[36]
Carriebowlinol (**36**)	Related to *L. majuscula*/east side of Carrie Bow Cay, Belize	Antibacterial activity against eleven marine bacterial strains and inhibits growth of three harmful marine fungal species (*D. salina*, L. *thalassiae,* and *Fusarium* sp.)	[37]
Malyngamides D (**37**)–F (**39**), 4 (**42**), and B (**43**)	*L. majuscula*/various locations	**37** and **38**: mild antibiotic against *Mycobacterium smegmatis* and *B. subtilis* **39**: activity against *S. aureus* **42** and **43**: weak inhibition of mycobacterial growth	[38,39,40]
Lyngbic acid (**41**)	Various marine cyanobacterial strains/various locations	Antimicrobial activity against *S. aureus* and *B. subtilus*; active against *Mycobacterium tuberculosis* H37Rv; and inhibits the growth of pathogenic and saprophytic marine fungi (ecological function)	[37,39,40,41]
Pitipeptolide A (**44**), B (**45**), and F (**46**)	*L. majuscula*/Piti Bomb Holes, Guam	**44** and **45**: moderately antimycobacterial **46**: potent in the disk diffusion assay against *M. tuberculosis*	[42,43,44]
Pitiprolamide (**47**)	*L. majuscula*/Piti Bomb Holes, Guam	Weak antibacterial activity against *M. tuberculosis*	[45]
Hormothamnins	*Hormothamnion enteromorphoides*/Playa de Luquillo, Puerto Rico	**48**: weak antibacterial activity against *B. subtilis* and *P. aeruginosa*; and antimicrobial against *B. subtilis* and *C. albicans* Hormothamnins A’, C/D, G, G’, G’’, J and K display antibacterial and antifungal activities	[46,47]
Malyngamide C (**51**)	*Stylocheilus longicauda*/Bush Key, Florida	Inhibits QS pathway in an LasR-based reporter gene assay without inhibiting bacterial growth	[48]
Laxaphycins A (**54**) and B (**55**)	*L. majuscula*, *Anabaena torulosa*/Moorea Atoll, French Polynesia	**54** and **55**: work synergistically against *C. albicans*	[49]
Lyngbyoic acid (**57**)	*L.* cf. *majuscula*/Indian River Lagoon and Dry Tortugas National Park, Florida	Effective against lasR; reduces pigment and elastase production; and antibiofilm activity	[50,51]
Benderadiene (**58**)	cf. *Lyngbya* sp./St. John’s Island, Singapore	Activity against *P. aeruginosa* PAO1 *lasB-gfp* and *rhlA-gfp*	[51]
Pitinoic acids A (**59**) and C (**61**)	*Lyngbya* sp./Piti Bay, Guam	**59**: inhibits QS in *P. aeruginosa* **61**: prevents induction of pro-inflammatory cytokine expression in LPS-induced THP-1 macrophages	[52]
Honaucins A (**62**)–C (**64**)	*Leptolyngbya crossbyana*/Hōnaunau Reef, Hawaii	**62**–**64**: QS inhibitors to *V. harveyi* BB120, and *E. coli* JB 525; inhibit lipopolysaccharide-stimulated nitric oxide production and repress the expression of pro-inflammatory cytokines in murine macrophages Synthetic **65** and **66**: effective anti-inflammatory compounds and exhibit improved inhibitory effects on QS activities	[53]
Tumonoic acids E (**69**)–I (**73**)	*Blennothrix cantharidosmum*/Duke of York Island, Papua New Guinea	**73**: moderate antimalarial activity **69**–**72**: inhibit QS systems against a wild-type strain of *V. harveyi*	[54]
8-*epi*-malyngamide C (**74**)	*L. majuscula*/Bush Key, Florida	Inhibits QS pathway in an LasR-based reporter gene assay without inhibiting bacterial growth	[48]
Trikoveramides A (**89**)–C (**91**)	*Symploca hydnoides*/Bintan	Moderate QS-inhibitory activities against *P. aeruginosa* PAO1 *lasB-gfp and rhlA-gfp* bioreporter strains	[55]
Trikoramide B (**93**)	*S. hydnoides*/Bintan	Inhibits PAO1 *lasB-gfp and rhlA-gfp*	[56]
Majusculoic acid (**96**)	Cyanobacterial mat/Sweetings Cay, Bahamas	Exhibits antifungal properties against *C. albicans* ATCC 14503 and *C. glabrata*	[57]
Kalkipyrones A (**100**) and B (**101**)	*Leptolyngbya* sp. and cf. *Schizothrix* sp./American Samoa and Panama	Toxicity against *Saccharomyces cerevisiae* ABC16-Monster strain	[58,59]
Amantelides A (**103**) and B (**104**)	Gray cyanobacterium (Oscilliatoriales)/Two Lover’s Point, Tumon Bay, Guam	**103**: broad spectrum of bioactivity against bacterial pathogens and marine fungi **104**: completely inhibits growth of *Dendryphiella salina*, but minimal effect on growth of *Lindra thalassiae* and *Fusarium* sp.	[60]
Swinholide-related molecules	*Geitlerinema* sp. and cf. *Phormidium* sp./Nosy Mitso-ankaraha Island, Madagascar, and American Samoa	**105**: antifungal activity	[61]
Dolastatins 10 (**108**) and 15 (**130**)	*Symploca* sp. VP642/Palau	**108** and **109** (synthetic): antifungal activity against several yeasts and filamentous fungi **108**, **130**, and synthetic **108** derivatives: antimalarial properties against *P. falciparum*	[61]
Majusculamide C (**110**) and 57-normajusculamide C (**111**)	*L. majuscula*/Enewetak Atoll, Marshall Islands	**110**: inhibits growth of fungal plant pathogens *Phytophthora infestans*, *Plasmopora viticola,* and *Rhizoctonia solani* **111**: Antimycotic properties against Saccharomyces pastorianus	[62,63]
Lyngbyabellin B (**112**) and hectochlorin (**113**)	*L. majuscula*/Dry Tortugas National Park, Florida, Hector Bay, Jamaica, and Boca del Drago Beach, Panama	**112** and **113**: antifungal activity against *C. albicans*	[64,65]
Lobocyclamides A (**115**)–C (**117**)	*L. confervoides*/Cay Lobos, Bahamas	Modest antifungal activity when tested against fluconazole-resistant fungi *C. albicans* and *C. glabrata*; mixtures of **115** and **116** in 1:1 ratio show synergistic antifungal activity	[66]
Hierridins A (**119**) and B (**118**)	*Phormidium ectocarpi*/red-pigmented cyanobacterial strain isolated from green algae, *Udothea petiolate*, from the coast of Mallorca	Mixture of **119** and **118** exhibits antiplasmodial activity against *P. falciparum* D6 and W2	[67]
Malyngolide dimer (**120**)	*L. majuscula*/Coiba National Park, Panama	Moderate antimalarial activity against chloroquine-resistant *P. falciparum* (W2)	[68]
Biselyngbyaside (**121**) and biselyngbyolide B (**122**)	*Lyngbya* sp./Okinawa	**121**: antimalarial activity against *P. falciparum* chloroquine-resistant K1 and chloroquine-sensitive FCR3 strains **122**: weaker antimalarial activities	[69]
Bastimolides A (**126**), B (**127**), and palstimolide A (**129**)	*Okeania hirsute* and *Leptolyngbya* sp./Isla Bastimentos Park, Panama, and Palmyra Atoll	**126**: potent activity against four multidrug-resistant strains of *P. falciparum*, including TM90-C2A, TM90-C2B, W2, and TM91-C235 **127**: strong antimalarial activity against CQ-sensitive *P. falciparum* strain HB3 **129**: potent antimalarial activity against the blood stage of *P. falciparum* Dd2 strain; active against intracellular *L. donovani* parasite infecting murine macrophage cells	[69]
Carmaphycins A (**138**) and B (**139**)	*Symploca* sp./off an anchor rope by a snorkeler south of the CARMABI research station, Curacao	**139**: potent activity against the asexual, liver, and sexual stages of *P. falciparum*	[70]
Dragomabin (**141**), dragonamide B (**143**), carmabin A (**144**), and dragonamide A (**142**)	*L. majuscula*/Panama	**141**, **142**, and **144**: moderate against the W2 chloroquine-resistant malaria strain	[71,72,73]
Ikoamide (**147**)	*Okeania* sp./Iko-pier, Kuroshima Island, Okinawa	Strong antiplasmodial activity against the asexual erythrocytic stage of the *P. falciparum* 3D7 clone	[74]
Mabuniamide (**148**)	*Okeania* sp./Odo, Okinawa	**148** and synthetic **149**: antiplasmodial activity	[75]
Hoshinoamides A (**150**)–C (**152**)	*Caldora penicillata*/Hoshino and Ikei Island, Okinawa	Exhibit antiplasmodial activity	[76,77]
Pemuchiamides A (**154**) and B (**155**)	*Hormoscilla* sp./Pemuchi Beach, Hateruma Island, Japan	**154**: strong growth-inhibitory activity against *T. brucei rhodesiense*	[78]
Kulolide-1 (**156**)	*Philinopsis speciosa*/Shark’s Cove, Pupukea, Oahu	Antimalarial activity against two malarial strains—*P. falciparum* Dd2 clone and 3D7 clone	[79]
Dudawalamides A (**157**)–D (**160**)	*Moorena producens*/Dudawali Bay, Papua New Guinea	**157** and **160**: exhibit the strongest activity against *P. falciparum* **160**: potent against *L. donovani*	[80]
Lyngbyabellins	*Okeania* sp. and *M. bouillonii*/Algetah Alkabira reef near Jeddah, Saudi Arabia, and Sabah, Malaysia	**161** and **162**: potent against *P. falciparum* strain FCR-3 **163**: moderately active against *P. falciparum* strain FCR-3	[81,82]
Kakeromamide B (**164**), ulongamide A (**165**), 18*E*-lyngbyaloside C (**166**), and lyngbyaloside (**167**)	*Moorena producens*/Fiji	**164** and **165**: moderate activity against *P. falciparum* blood stages **164**, **166**, and **167**: moderate liver-stage antimalarial activity against *P. berghei* liver schizonts	[82]
Veraguamides M (**168**) and N (**169**)	*Lyngbya* sp./Coiba National Park, Panama	**168** and **169**: active against *P. falciparum* **169**: active against *Leishmania donovani*	[83]
Companeramides A (**170**) and B (**171**)	marine cyanobacterial assemblage/Coiba Island, Panama	Active against three strains of the malaria parasite *P. falciparum* using a fluorescence-based assay	[84]
Wajeepeptin (**172**)	*Moorena* sp./Wajee Coast, Ie Island, Okinawa	Potent antitrypanosomal activity against *T. brucei rhodesiense*	[85]
Venturamides A (**173**) and B (**174**)	*Oscillatoria* sp./Buenaventura Bay, Portobelo National Marine Park, Panama	Strong activity against *P. falciparum*; mild activity against *T. cruzi* and *L. donovani*	[86]
Lagunamides A (**175**)–C (**177**)	*L. majuscula*/Pulau Hantu, Singapore	Potent activity against the *P. falciparum* NF54 strain	[87,88]
Symplocamide A (**178**)	*Symploca* sp./Sunday Island, Papua New Guinea	Significant antimalarial activity against W2 *P. falciparum*; moderate activity against *T. cruzi* and *L. donovani*	[89]
Kagimminols A (**179**) and B (**180**)	*Okeania* sp./Kagimmi Beach, Okinawa	Moderate selective growth-inhibitory activity against *Trypanosoma brucei rhodesiense* strain IL-1501	[90]
Coibacins A (**181**)–D (**184**)	cf. *Oscillatoria* sp./near Uvas Island, Coiba National Park	**181**: potent activity against *L. donovani* and *L. mexicana* axenic amastigotes	[91]
Bromoiesol sulfates A (**185**), B (**186**) and hydrolysates (**187** and **188**)	*Salileptolyngbya* sp./Ie-Island, Okinawa	**187** and **188**: antitrypanosomal activity against *T. brucei rhodesience* IL-1501 strain	[92]
Akunolides A (**189**)–D (**192**), polycavernoside E (**193**)	*Okeania* sp./Akuna Beach, Okinawa	**189**–**193**: moderate antitrypanosomal activities against *T. brucei rhodesiense*	[93,94]
Hennaminal (**194**) and hennamide (**195**)	*Rivularia* sp./Higashihennazaki, Miyako Island, Okinawa	Moderate growth-inhibitory activity against the bloodstream form of *Trypanosoma brucei rhodesiense*	[95]
Hoshinolactam (**197**)	Marine cyanobacterium/Hoshino, Okinawa	Potent antitrypanosomal activity against the *Trypanosoma brucei brucei* GUTat 3.1 strain	[96]
Beru’amide (**198**)	*Okeania* sp./Beru, Kasari-cho, Kagoshima, Japan	Potent antitrypanosomal activity against *T. brucei rhodesiense*	[97]
Iheyamides A (**201**)–C (**203**) and iheyanone (**204**)	*Dapis* sp./Noho Island, Okinawa	**201**: moderate antitrypanosomal activity against *T. brucei rhodesiense* and *T. brucei brucei* **204**: exhibits antitrypanosomal activity	[98,99]
Kinenzoline (**205**)	*Salileptolyngbya* sp./Kinenhama Beach, Kagoshima, Japan	Moderate growth-inhibitory activity against *T. b. rhodesiense*	[100]
Dragonamides A (**142**), E (**206**), and herbamide B (**207**)	*L. majuscula*/Bastimentos National Park, Bocas del Toro, Panama	Demonstrates antileishmanial activity	[101]
Almiramides A (**208**)–C (**210**)	*L. majuscula*/from mangrove roots in the Bocas del Toro National Marine Park, Panama	**209** and **210**: strong activity against *L. donovani* Synthetic analogs (e.g., **211**–**215**, **219**–**221**): improved antiparasitic activity	[102,103,104]
Viridamides A (**222**) and B (**223**)	*Oscillatoria nigro-viridis*/Panama	**222**: significant activity against *T. cruzi*, *L. Mexicana,* and *P. falciparum*	[105]
Amantamide C (**224**)	*Okeania* sp./Tonaki Island, Japan	Inhibits the growth of *T. brucei rhodesiense*	[106]
Okeaniazole A (**225**)	*Okeania hirsute*/Kuba Beach, Nakagusuku, Okinawa	Inhibitory activity against *Leishmania major*	[19]
Janadolide (**226**)	*Okeania* sp./Janado, Okinawa	**226**: potent antitrypanosomal activity against *Trypanosoma brucei brucei* GUTat 3.1 strain Several simplified analogs (e.g., **227**): moderate micromolar-range antitrypanosomal activity against *T. brucei rhodesiense* and *T. cruzi*	[107,108]
Motobamide (**228**)	*Leptolyngbya* sp./Bise, Okinawa	Inhibits the growth of the bloodstream form of *T. b. rhodesiense*	[109]
Barbamide (**229**)	*L. majuscula*/Barbara Beach, Curacao	Molluscicidal activity against *B. glabrata*	[110]
Cyanolide A (**230**)	*L. bouillonii*/shallow reef wall outside Pigeon Island, Papua New Guinea	Molluscicidal activity against *B. glabrata*	[111]
Thiopalmyrone (**231**) and palmyrrolinone (**232**)	cf. *Oscillatoria* and *Hormoscilla* sp./Palmyra Atoll	Molluscicidal activity against *B. glabrata*	[112]
